# Salinity changes the nitrification activity and community composition of comammox *Nitrospira* in intertidal sediments of Yangtze River estuary

**DOI:** 10.1128/msystems.01026-22

**Published:** 2023-06-12

**Authors:** Ran Jiang, Wen-Jing Qin, Ru-Yi Zhang, Kai Zhang, Xing Huang, Yong Li, Chen-Hao Zhou, Ting Zhu, Yan Zhang, Bin Zou, Ming Nie, Sung-Keun Rhee, Zhe-Xue Quan

**Affiliations:** 1 Ministry of Education Key Laboratory for Biodiversity Science and Ecological Engineering, National Observations and Research Station for Wetland Ecosystems of the Yangtze Estuary, Institute of Biodiversity Science and Institute of Eco-Chongming, School of Life Sciences, Fudan University, Shanghai, China; 2 Zhejiang Provincial Key Laboratory of Agricultural Resources and Environment, College of Environmental and Resource Sciences, Zhejiang University, Hangzhou, China; 3 Department of Microbiology, Chungbuk National University, Cheongju, Republic of Korea; E O Lawrence Berkeley National Laboratory, Berkeley, California, USA

**Keywords:** comammox *Nitrospira*, salinity, intertidal sediment, DNA-SIP, potential ammonium oxidation rate

## Abstract

The newly discovered complete ammonia-oxidizing (comammox) *Nitrospira* has been identified in different environments, including coastal environments, where salinity is one of the most important factors for the abundance and activity of nitrifiers. Here, we demonstrate the effect of salinity on comammox *Nitrospira*, canonical ammonia-oxidizing bacteria (AOB), and ammonia-oxidizing archaea (AOA) in the intertidal sediments of the Yangtze River estuary based on microcosm experiments, DNA stable-isotope probing (DNA-SIP), and potential ammonium-oxidation rate (PAR) tests for different groups of ammonia oxidizers with selective inhibitors. During microcosm incubations, the abundance of comammox *Nitrospira* was more sensitive to increased salinity than that of other ammonia oxidizers. The results obtained with DNA-SIP heavy fractions showed that the dominant phylotype in clade A.2 (containing genes involved in the adaptation to haloalkaline environments) had high proportions in comammox *Nitrospira* community under both freshwater (0.06% salinity) and highly saline water (3% salinity) conditions. In contrast, another phylotype of clade A.2 (which lacks these genes) was dominant only under freshwater conditions. The PARs confirmed that comammox *Nitrospira* presented greater contributions to nitrification under freshwater conditions with a PAR of 4.37 ± 0.53 mg N·day^–1^·kg soil^–1^ (54%) than under saline water conditions with a PAR of 0.60 ± 0.94 mg N·day^–1^·kg soil^–1^ (18%). Moreover, AOA were specific to saline water conditions, whereas AOB were common under both freshwater and saline water conditions (44% and 52%, respectively). The present study provided evidence that salinity markedly affects the activity of comammox *Nitrospira,* and that the salt sensitivity of different phylotypes varies.

**IMPORTANCE:**

Complete ammonia oxidation (comammox) is a newly discovered type of nitrification through which ammonia is oxidized to nitrate in an organism. Comammox *Nitrospira* were abundantly found in coastal ecosystems and demonstrated high community diversity. Changes in salinity are considered one of the most important factors to comammox *Nitrospira* in coastal ecosystems; however, reports on the correlation between them remain inconsistent. Therefore, it is critical to experimentally determine the influence of salinity on comammox *Nitrospira* in the coastal ecosystem. This study demonstrated a clear effect of salinity on the abundance, activity, and relative contribution of different ammonia oxidizers, especially for comammox *Nitrospira*. To the best of our knowledge, this is the first study demonstrating comammox *Nitrospira* activity at seawater salinities, implying the existence of a salt-tolerant type comammox *Nitrospira*, despite its activity being much lower than in freshwater conditions. The indicated correlation between the activity of specific comammox *Nitrospira* and salinity is anticipated to provide insights into the distribution of comammox *Nitrospira* and their potential contributions in estuaries and coastal ecosystems.

## INTRODUCTION

Nitrification, a process whereby ammonia is converted to nitrate, plays a key role in global nitrogen cycling (1). Over the past century, nitrification has been exclusively considered a two-step microbial process involving two different types of microbes. The first step is ammonia oxidation, which was thought to be performed by ammonia-oxidizing bacteria (AOB) and ammonia-oxidizing archaea (AOA), while the second is nitrite oxidation, which was thought to be performed by nitrite-oxidizing bacteria (NOB) (2). However, the recent discovery of complete ammonia-oxidizing (comammox) bacteria, single microbes that perform both ammonia and nitrite oxidation, has challenged the century-old paradigm and redefined key processes in the biogeochemical nitrogen cycle (3, 4). All reported comammox bacteria belong to sublineage II of the *Nitrospira* genus (3 - 5). Based on phylogenetic analysis of the *amoA* gene (which encodes the α-subunit of ammonia monooxygenase, a common biomarker of ammonia oxidizers), comammox *Nitrospira* can be divided into clade A (including clades A.1 and A.2) and clade B (6, 7).

Several recent studies on aerobic ammonia oxidation employed quantitative polymerase chain reaction (qPCR) analysis or PCR-amplicon sequencing to investigate the absolute or relative abundances of ammonia oxidizers (including AOA, AOB, and comammox *Nitrospira*) in different environments (8 - 11). Generally, these studies also analyzed correlations between the abundance of ammonia oxidizers and different environmental factors (such as the pH, salinity, and temperature) (8, 11 - 13). Previously, microcosm-incubation experiments were used to investigate the impact of specific environmental factors on ammonia oxidizers (14, 15) or combined analysis with DNA stable-isotope probing (DNA-SIP) (16, 17). DNA-SIP can reveal the impact of environmental factors on the growth of autotrophic ammonia oxidizers over incubation periods lasting several days but does not directly determine the activities of ammonia oxidizers. Therefore, a method based on specific inhibitors, such as 1-octyne (an inhibitor of AOB) (18), is often used to separately detect the potential ammonium-oxidation rates (PARs) of AOA and AOB (19, 20). Additionally, a recent discovery revealing that chlorate could inhibit ammonia oxidation in comammox *Nitrospira* (21, 22) makes it possible to determine the individual activities of all three different types of ammonia oxidizers.

The intertidal zone, as a link between terrestrial and marine ecosystems, experiences fluctuations in salinity, temperature, and wave forcing (23). The intertidal zone is the most productive component of coastal ecosystems and is recognized for its importance in the biogeochemical cycling of nitrogen (24), with unique phases of coupling aerobic and anaerobic sedimentary microenvironments for nitrification and denitrification processes (25). Salinity changes are important environmental factors in decreasing potential nitrification rates and reshaping the community structure of canonical ammonia oxidizers in coastal sediments (26 - 29). Previous studies have demonstrated that the recently discovered comammox *Nitrospira* is broadly distributed in the coastal ecosystem (including intertidal sediments) and that the proportion of comammox *Nitrospira* (compared to AOA and AOB) varied at different sampling sites (8, 12, 30) based on qPCR analysis and sequencing PCR-amplicon libraries. Previous research also revealed significant correlations between comammox *Nitrospira* and salinity, where some correlations were negative (13, 30), while others were positive (8, 12). Additionally, previous analyses using metagenomic data from public databases have revealed relatively high proportions of comammox *Nitrospira* (compared with AOA and AOB) in coastal ecosystems (6), suggesting that comammox *Nitrospira* could potentially be important nitrifiers in this ecosystem, even though these high proportions were not observed in most qPCR-based studies, possibly due to differences in sampling sites and insufficient coverage of these comammox *Nitrospira* primer sets (31, 32). Therefore, as a key factor for comammox *Nitrospira* in the coastal ecosystem, the impact of salinity on their abundance, activity, and phylotype is worth investigating.

In this study, we conducted microcosm-incubation experiments with intertidal sediments containing three ammonia oxidizers with approximately equal abundance, which was determined using qPCR of *amoA* genes from different ammonia oxidizers. The aims of the study are to: (i) assess the effect of salinity on the abundance of nitrifiers, especially for comammox *Nitrospira*; (ii) identify active comammox *Nitrospira* under freshwater (0.06% salinity) and saline water (3% salinity) conditions through amplicon and metagenome sequencing of DNA-SIP samples; and (iii) distinguish the PAR of comammox *Nitrospira* from those of other ammonia oxidizers using specific inhibitors under freshwater and saline water conditions.

## MATERIALS AND METHODS

### Sampling

A surface sediment sample (above 5 cm) was collected by shovel from a low-tide coastal flat of the Yangtze River in Chongming, Shanghai, China (121.67°E, 31.69°N) in June 2019. The sediment was covered with *Phragmites australis* and had a very thin layer of water (at low tide). Samples were collected, placed in a sterile plastic bag, and transported to our laboratory with an ice pack. The sediment sample was then passed through a 1-mm sieve (to remove stones or roots), homogenized, and stored at 4°C for 6 months. The pH and salinity of the sediment were 8.35 and 5.28 g·kg dry soil^−1^ (0.33%), respectively, as determined using SevenExcellence Meters (Mettler Toledo, Switzerland) with soil-water suspensions (1:2.5 soil/water), shaken for 30 min. The water content of the soil was 58%. The ammonium content was 1.26 mg N·kg soil^−1^, as measured by the modified indophenol method, which is based on the well-established Berthelot reaction (33).

### Sediment-microcosm incubations

**Fig 1:**
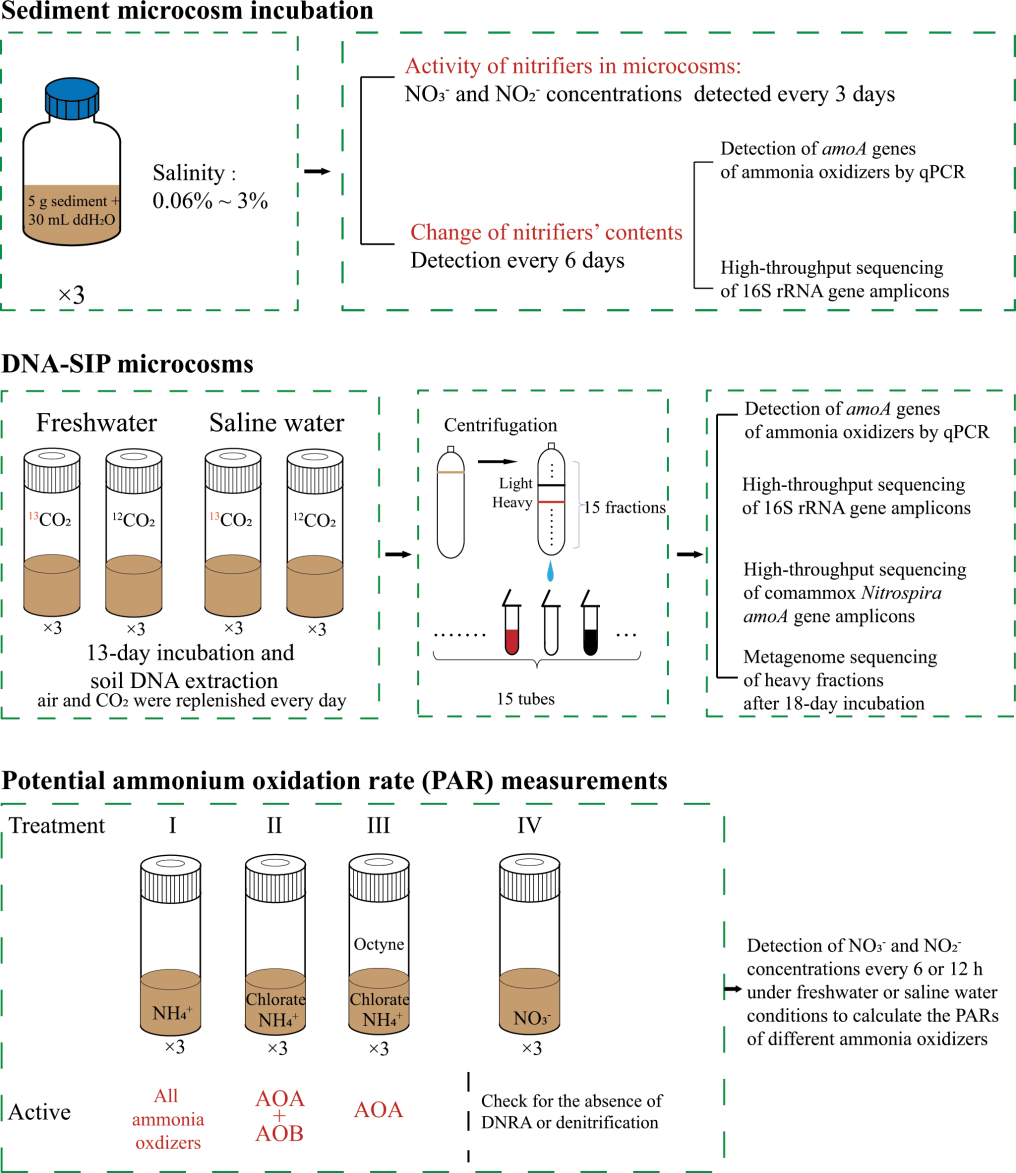
Experimental design of this study. Abbreviations: AOA, ammonia-oxidizing archaea; AOB, ammonia-oxidizing bacteria; DNRA, dissimilatory nitrate reduction to ammonium.

A flowchart of the main steps followed in this study is shown in Fig. 1. Considering that the salinity of Yangtze River estuary sediments varies during different seasons (34), the microcosms were constructed with 0.06%, 0.5%, 1%, 1.5%, or 3% salinity, to examine the effects of salinity on nitrifiers in sampled intertidal sediments. The salinity was adjusted with NaCl. Considering that the intertidal sediment is regularly covered with water (partially exposed to air when we sampled it at low tide) and suspended by tides, the incubation was performed in 100 mL glass vials containing 5 g sieved sediment and 30 mL ddH_2_O with 0.1 mM urea (amended daily except on day 2, as the ammonium content at the sample site was 0.09 mM), with a low stirring speed (approximately 100 rpm). The microcosms were incubated in the dark at 28°C (according to the temperature of the sampled days) for 18 days. About 1 mL of the solution was removed from each vial every 3 days and centrifuged at 10,000×*g* for 1 min to separate the precipitate (stored at −20°C for molecular analysis) and supernatant (filtered by 0.22 mm syringe filters and stored for up to 3 days at 4°C until ion analysis). The NO_3_^−^ and NO_2_^−^ concentrations in the supernatants were determined using a Dionex ICS-1100 ion chromatography column (Thermo Fisher Scientific, Waltham, MA, USA). The contents and populations of nitrifiers in the precipitates were detected at 6-day intervals using qPCR and 16S rRNA gene amplicons sequencing.

### DNA-SIP microcosm incubations

DNA-SIP microcosms were constructed to identify active nitrifiers, especially comammox *Nitrospira*, under freshwater or saline water conditions (Fig. 1). Briefly, 5 g of sieved sediment, 30 mL of 20 mM 4-morpholineethanesulfonic acid buffer (to maintain pH around 8.35 after CO_2_ addition), 0.1 mM ammonium (added every day except on day 2) were mixed and incubated using a magnetic rotor in 110 mL glass vials, in the dark for 13 days at 28°C. The magnetic rotor was set at a low speed (approximately 100 rpm). Two treatments with 1% ^12^CO_2_ or ^13^CO_2_ were performed in the headspace of these vials in triplicate microcosms under freshwater and saline water conditions. To maintain aerobic conditions, the headspaces of the vials (including the 1% CO_2_) were replenished daily. We purchased ^13^CO_2_ (99 atom% ^13^C) from the Shanghai Engineering Research Center of Stable Isotopes (Shanghai, China). Samples were destructively collected on day 13 and immediately frozen at −20°C for subsequent molecular analysis.

Owing to the relatively high content of comammox *Nitrospira* in the heavy fraction with fresh water in 13-day DNA-SIP microcosms, the subsequently conducted further microcosms with fresh water, which were incubated for 18 days to increase the content of comammox *Nitrospira*, were used to obtain metagenome-assembled genomes (MAGs) of active comammox *Nitrospira*.

### DNA extraction and SIP-based fractionation

DNA was extracted from 0.25 g of the original sediment or approximately 0.16 g of microcosm sediments (precipitates from 1 mL mixtures) using a DNeasy PowerSoil Kit (Qiagen, Dusseldorf, Germany) according to the manufacturer’s instructions.

SIP fractionation was performed as previously described (35). Briefly, approximately 3.0 µg of DNA from each sample was mixed with a CsCl solution to a buoyant density of 1.725 g/mL before ultracentrifugation. To avoid bubble formation, each mixture was dropped slowly into a 5.1-mL Beckman ultracentrifuge tube (Beckman Coulter, Palo Alto, CA, USA) with an injector. Isopycnic density ultracentrifugation was performed at 177,000 × *g* for 44 h at 20°C with a Vti65.2 vertical rotor. After ultracentrifugation, 14–16 DNA fractions were obtained by displacing the gradient medium with sterile water (containing bromophenol blue dye) from the top of the ultracentrifuge tube, using a syringe pump (New Era Pump Systems Inc., Farmingdale, NY, USA) at a flow rate of 0.34 mL/min. The volume of each fraction was approximately 340 µL, and the refractive index was determined using a Brix/RI-Check digital handheld refractometer (Reichert, Inc., Buffalo, NY, USA). The fractionated DNA was purified using PEG-6000 and 70% alcohol, and dissolved in 30 µL TE buffer, as previously described (36). The purified DNA was subjected to qPCR analysis, 16S rRNA and comammox *Nitrospira amoA* gene amplicons sequencing, and metagenome sequencing.

### qPCR-based detection of the *amoA* gene

qPCR was performed using the Mx3000P real-time PCR system (Stratagene, Bellingham, WA, USA) and FastStart Universal SYBR Green Master Mix (Rox) (Roche, Mannheim, Germany) with the following thermocycling protocol: 95°C for 10 min, followed by 40 cycles of 95°C for 30 s, 55°C for 30 s, and 72°C for 30 s. The abundance of the *amoA* genes of AOA, AOB, and comammox *Nitrospira* clades A and B were quantified using the Arch-amoAF/Arch-amoAR, amoA1F/2R, CA377f/C576r, and CB377f/C576r primer sets, respectively (Table S1). For each assay, triplicate standard curves were generated using 10-fold serially diluted plasmids (approximately 10^1^–10^7^ gene copies/μL) containing the target genes (32). The qPCR results were analyzed using MxPro qPCR software (version 3.0; Agilent Technologies, Santa Clara, CA, USA). Melting curves were analyzed to determine the effects of primer dimers. The consistency of the results was confirmed by observing the linear relationship between the threshold cycle and the log value of the gene copy number. The amplification efficiencies of all *amoA* genes were 83%–94%, and the *r^2^* values were >0.99.

### PCR and high-throughput sequencing of the 16S rRNA and comammox *Nitrospira amoA* genes

DNA from the original sediment sample and incubated microcosm samples were amplified using the 341F/806R primer set (targeted prokaryotic 16S rRNA gene), and different DNA-SIP fractions were amplified using the 515F/806R primer set (Table S1). The following thermocycling program was used for 16S rRNA gene amplification: an initial step at 95°C for 5 min, followed by 35 cycles at 95°C for 30 s, 55°C for 45 s (515F/806R) or 60 s (341F/806R), and 72°C for 45 s (515F/806R) or 60 s (341F/806R), with a final step at 72°C for 10 min. Comammox *Nitrospira amoA* gene sequences were analyzed using partial nested PCR with the DNA-SIP samples, using the A189Y/C576r and CA209F/C576r primer sets (Table S1). Partially nested PCR amplification was performed as described previously (6). All PCR products were prepared for Illumina HiSeq sequencing.

### High-throughput sequencing analysis of amplicons

Sequences were primarily processed using the QIIME software package (version 1.9.1) (37). The barcode sequence region (12 bases for all amplicons) was removed, and amplicon sequences with base qualities exceeding Q20 were retained using sickle software (version 1.33; https://github.com/najoshi/sickle). Quality-controlled sequences from both directions were assembled into single sequences. Sequences were assigned to individual samples according to their barcodes and checked for chimeric sequences using USEARCH software (version 61) based on the 16S rRNA gene (38) and copper-containing membrane-bound monooxygenase gene (6) reference sequences.

The sequences of amplified prokaryotic 16S rRNA genes or the comammox *Nitrospira amoA* gene were assigned to operational taxonomic units (OTUs) according to 97% or 90% identity thresholds, respectively, and sequences shorter than 150 base pairs (bp; 515F/806R), 300 bp (341F/806R), or 250 bp (CA209F/C576r) were removed using QIIME. Representative sequences of the comammox *Nitrospira amoA* gene were translated into protein sequences using FrameBot (39). The Silva SSU nr132 database was used as a reference for 16S rRNA gene classification with QIIME.

The obtained OTUs of AmoA amino acid sequences of comammox *Nitrospira* or 16S rRNA gene sequences of AOB or AOA were separately aligned with their reference sequences using MUSCLE or CLUSTALW in MEGA X (40). Phylogenetic trees were constructed using the maximum-likelihood method with 1,000 bootstrap replicates and visualized with their relative abundances using table2itol.R (https://github.com/mgoeker/table2itol) and iTOL (https://itol.embl.de/). OTUs were compressed into a single OTU if a branch did not contain reference sequences.

### DNA-SIP-based metagenome analysis of *Nitrospira*

Metagenome libraries were generated from DNA-SIP fractions (7 - 9) from an 18-day microcosm (under freshwater conditions and treated by ^13^CO_2_) using the VAHTS Universal DNA Library Prep Kit for Illumina V3 (Vazyme Biotech, Nanjing, China), according to the manufacturer’s instructions. The libraries were sequenced on an Illumina Novaseq 6000 150 bp paired-end platform at the Novogene facility in Tianjin. Finally, about 20 Gb of data was acquired for each fraction. Quality-control analysis was performed using FastQC software (version 0.10.1; http://www.bioinformatics.babraham.ac.uk/projects/fastqc/) and trimmomatic software (41). Only high-quality paired reads were used for subsequent analysis.

Two assembly strategies were used to obtain MAGs with high completeness. In one strategy, a separate assembly was prepared for each fraction. Using this strategy, DNA from three fractions was individually assembled using the SPAdes software (version 3.14.1) (42) in meta mode. Sequences were then mapped using Burrow-Wheeler Aligner (BWA) (v2.12.1) (43). Samtools (version 1.9) (44) was used to convert the sam to bam format. The scaffold depths were calculated using the jgi_summarize_bam_contig_depths command of MetaBAT2 (45), whereas the scaffolds were selected using the parameters minContigLength 1000 and minContigDepth 2. Selected scaffolds were binned using MetaBAT2, and all binned MAGs were classified using GTDB-tk (version 1.3.0) (46). The completeness and contamination of MAGs belonging to the *Nitrospira* genus were evaluated using checkM software (version 1.0.18) (47).

Since these three DNA fractions were separated from one sample, they may comprise the same microbes, although the relative microbial abundances differed in the three fractions. Thus, our second strategy involved co-assembly. Paired reads from the three fractions were combined and assembled using the default parameters of MegaHit (version 1.2.7) (48). The reads of each fraction were then mapped to the assembled scaffolds. Sequencing depth information from all three fractions was used to bin the scaffolds, as described above. MAGs obtained using both strategies were deduplicated using dRep (49).

Gene calling was performed using Prodigal software (version 2.6366) in meta mode (50). To obtain the *amoCAB* gene sequence from *Nitrospira* bin98 (which could not be retrieved using either assembly strategy), *Nitrospira* bin98 was reassembled by the recruited reads on reference genome of *Candidatus* Nitrospira kreftii due to the high average nucleotide identity between them. BLASTn was performed to recruit quality-controlled sequences from each fraction, using sequences with 95% identity, and the hit reads were retrieved using the filterbyname.sh command of the BBMap tool kit (sourceforge.net/projects/bbmap/). These hit sequences were assembled in meta mode using SPAdes. Annotation was conducted using Kofamscan (51). For *Nitrospira* bin457, ContigExtender (52) was used to extend the incomplete *amoA* gene sequence of *Nitrospira* bin457. The accuracy of the extended *amoA* gene sequence was checked using the mapping results of BWA.

Forty-four *Nitrospira* and five outgroup genomes or MAGs were downloaded from the National Center of Biotechnology Information (https://www.ncbi.nlm.nih.gov/assembly) to generate a *Nitrospira* genome reference data set. *Nitrospira* MAGs assembled in this study and the reference *Nitrospira* genome data set were imported into Anvi'o software (version 6.2) (53) using anvi-script-FASTA-to-contigs-db. Seventy-one bacterial housekeeping genes were extracted using anvi-get-sequences-for-hmm-hits. The housekeeping genes were aligned using MUSCLE (54). A maximum-likelihood phylogenetic tree was constructed for *Nitrospira* using RAxML (55) with 500 bootstrap replicates and visualized using iTOL (https://itol.embl.de).

The *amoA* gene sequences of different ammonia oxidizers were retrieved from the metagenome data generated in this study, as reported previously (6) with an updated data set (https://github.com/traminer23333/updateCuMMOdataset) for Framebot.

Putative orthologous genes related to high salinity adaptation in *Nitrospira* genus from this study were predicted through OrthoFinder (https://github.com/davidemms/OrthoFinder) by comparing with other representative genomes of isolated and metagenomic source *Nitrospira*.

### Measuring PARs of different ammonia oxidizers

The incubation content and condition of potential ammonium oxidation rate measurement experiments were carried out as DNA-SIP microcosm incubations. Ammonia was added 2 h after adding different inhibitors, where the ammonia addition step was considered the initial time. Four treatments (treatments I–IV) with three replicates were performed for each salinity level (Fig. 1). In the no-inhibitor group (treatment I): 0.1 mM ammonium chloride was added to the vials, and the headspace was replenished every day. The chlorate-only group (treatment II) was treated the same as treatment I, except that 1 mM chlorate was added. With the chlorate and 1-octyne group (treatment III), 0.1 mM ammonium chloride, 4 µM 1-octyne, and 1 mM chlorate were added to the vials, and the headspace (including 1-octyne) was replenished every day. In the nitrate group (treatment IV), 0.1 mM nitrate was added to determine the variation caused by DNRA and denitrification during aerobic treatments I–III. Samples with the same salinity were taken at equal time intervals for at least five time points. The NO_2_^−^ and NO_3_^−^ concentrations were determined by photometry based on the VCl_3_/Griess method (33), except for NO_3_^−^ concentrations in treatments II and III, which were determined by Dionex ICS-1100 ion chromatography because chlorate obtained electrons from VCl_3_ and became bleached hypochlorite, which would invalidate the photometric results.

During treatment I (no added inhibitors), ammonia was oxidized by AOA, AOB, and comammox *Nitrospira*. The PAR of treatment I was equal to the sum of the PAR of AOA, AOB, and comammox *Nitrospira*. During treatment II, 1 mM chlorate was added since it could completely inhibit ammonia oxidation and nitrite production activities of *Nitrospira inopinata*, while chlorate did not inhibit either of the tested AOA or AOB strains (22). Ammonia oxidation in treatment II was carried out by AOA and AOB. Hence, the PAR of comammox *Nitrospira* was calculated as the PAR of treatment I minus that of treatment II. During treatment III, chlorate and 1-octyne were added since previous studies have reported that 4 µM of 1-octyne can completely inhibit AOB, and AOA was unaffected by up to 20 µM at least 20 h (18). Therefore, in treatment III, comammox *Nitrospira*, strict NOB, and AOB were inhibited. Nitrite production during treatment III was only attributed to ammonia oxidation by AOA; therefore, the PAR of treatment III was considered as the PAR of AOA. The PAR of AOB was calculated as the PAR of treatment II minus that of treatment III.

### Statistical analysis

All analyses were performed using R software (version 3.6.1). Bar plots for the *amoA* of ammonia oxidizers or 16S rRNA gene of nitrifiers during microcosm incubation were drawn using ggplot (ggplot2 package, version 3.2.1). Duncan’s test (agricolae package, version 1.3–3) was conducted to determine the differences in the abundance of ammonia oxidizers across the salinity gradient. Linear-regression analysis of the PARs was performed using the lm () function. Connected scatterplots for the *amoA* genes of ammonia oxidizers in different fractions of DNA-SIP microcosms were drawn using ggplot (ggplot2 package, version 3.2.1). We used gggenes (version 0.4.1) to draw diagrams to compare the salt-tolerance genes of *Nitrospira kreftii* and our two MAGs.

## RESULTS

### Effect of salinity on the growth of ammonia oxidizers

Treatments with five different salinities (i.e., 0.06%, 0.5%, 1%, 1.5%, and 3% NaCl), reflecting conditions ranging from freshwater (<0.1% salinity) to saline water (3% salinity), were designed to assess the impact of salinity on the abundance of ammonia oxidizers in microcosms.

The changes in NO_2_^−^ and NO_3_^−^ concentrations were measured every 3 days during the 18-day microcosm incubations (Fig. S1). The concentrations of accumulated NO_3_^−^ were similar in the microcosms at different salinity levels (Fig. S1a) and matched the stoichiometric conversion. NO_2_^−^ transiently accumulated, while the degree of accumulation increased with increasing salinity, indicating that the nitrite oxidation process in this sample was more sensitive than ammonia oxidation to salinity (Fig. S1b). The NO_2_^−^ concentration ranged from approximately 0.025 to 0.1 mM on days 3–12 under 0.5%–3% salinity.

**Fig 2:**
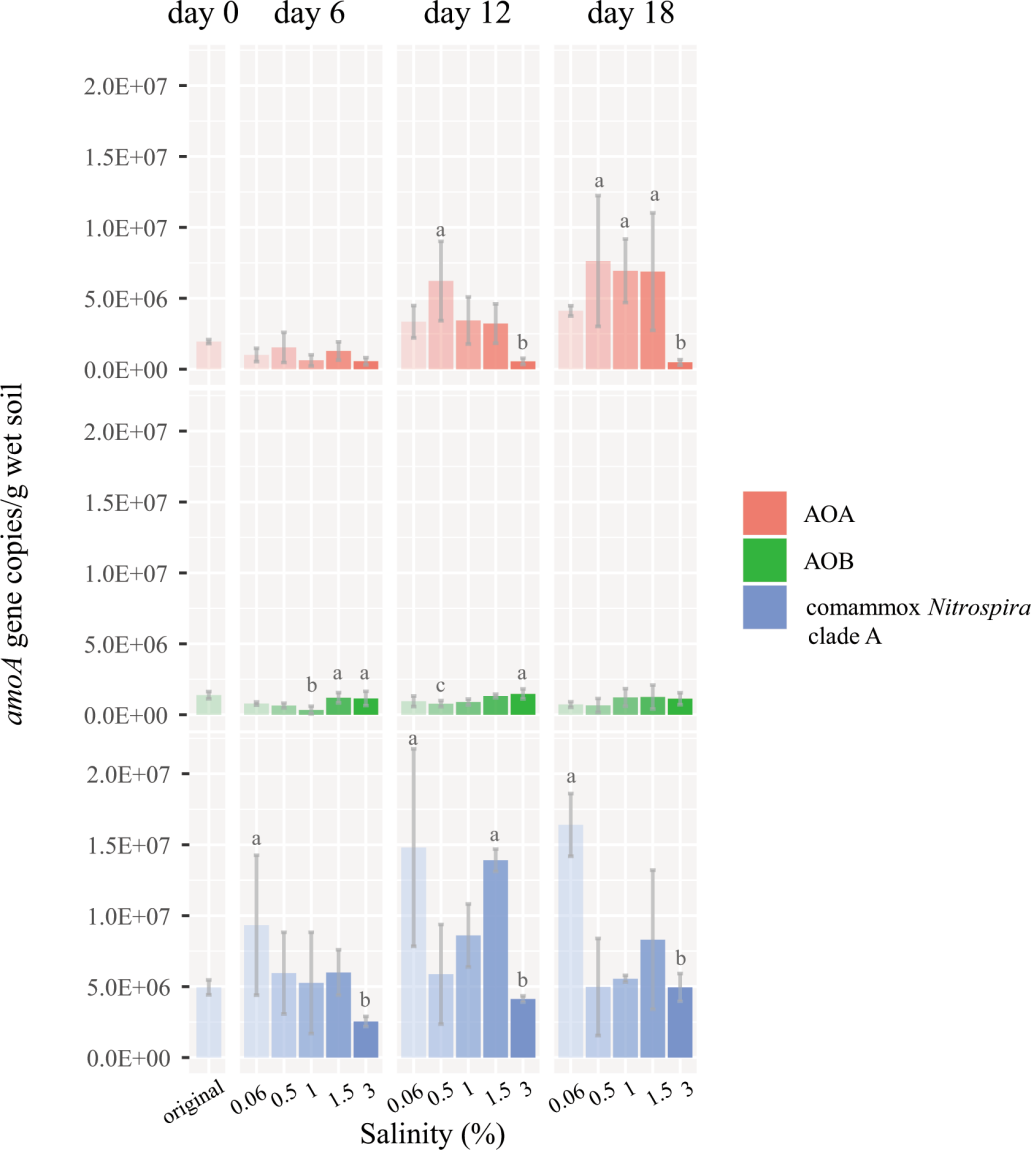
Changes in the *amoA* gene abundance of AOA, canonical AOB, and comammox *Nitrospira* clade A. Microcosms were incubated for 18 days under different salinity conditions. The error bars indicate the standard errors of triplicate samples. The transparency of the bars indicates the salinity of the microcosms. Different letters represent significant differences. Abbreviations: AOA, ammonia-oxidizing archaea; AOB, ammonia-oxidizing bacteria.

Changes in the compositions of ammonia oxidizers in the microcosms were monitored at 6-day intervals based on qPCR analysis of the *amoA* gene and high-throughput sequencing of prokaryotic 16S rRNA gene amplicons. The qPCR results showed that the *amoA* gene of comammox *Nitrospira* clade A was most abundant in freshwater microcosms (0.06% salinity) and significantly higher than in saline water microcosms (3% salinity) during the 18 days of incubation (Fig. 2). Compared with the original sediment, comammox *Nitrospira* clade A was threefold more abundant in freshwater microcosms after 18 days of incubation (increasing from 5×10^6^ to 1.5×10^7^ copies/g wet soil) but barely changed in the saline water microcosms. Comammox clade B was not detected in the original intertidal sediment samples using qPCR and partial nested PCR analysis of the comammox *Nitrospira amoA* gene (data not shown); since the low abundance of clade B has been previously reported in intertidal sediments (8, 12, 13), clade B was not quantified during the following microcosm incubation and DNA-SIP experiments. The abundance of the AOB *amoA* gene was higher under saline water conditions than under freshwater conditions during the 18-day incubation period. The AOA abundance was higher in the mesohaline (0.5% salinity) microcosms than in the saline water microcosms during the incubation period. The 16S rRNA gene sequencing results showed that the relative abundance of AOB, especially with the *Nitrosomonas* genus, increased with salinity and surpassed that of AOA after 18 days of incubation in the saline water microcosms, whereas AOA (*Nitrosoarchaeum* and *Nitrosopumilus*) exhibited an opposite trend (Fig. S2). Regarding the *Nitrospira* genus (including comammox bacteria and strict NOB), 16S rRNA gene sequencing results revealed that it was more abundant under freshwater conditions than under saline water conditions (Fig. S3). The proportion of other NOBs was much lower than that of *Nitrospira*. The main genus of other NOBs was *Nitrotoga* in most microcosms, but *Nitrospina* in saline water microcosms.

### DNA-SIP in different salinity microcosms

**Fig 3:**
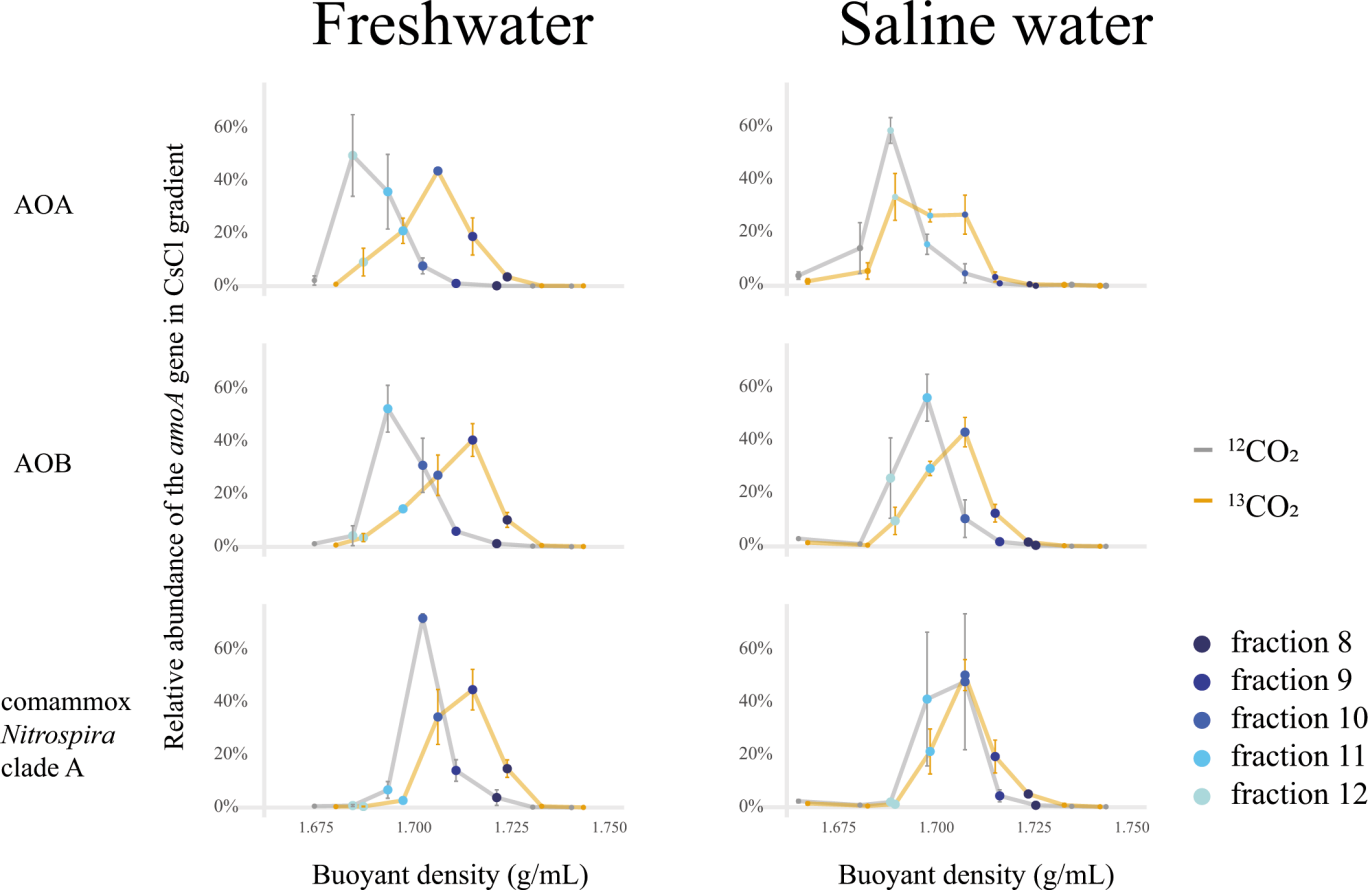
Distributions of the relative abundances of AOA, AOB, and comammox *Nitrospira* clade A *amoA* genes. The *amoA* genes were detected in different fractions from DNA-SIP microcosms after treatment with ^12^CO_2_ or ^13^CO_2_ for 13 days under freshwater (0.06% salinity) or saline water (3% salinity) conditions. The error bars represent the standard errors from three replicate incubations.

DNA-SIP microcosms were incubated for 13 days under freshwater and saline water conditions to identify functionally active ammonia oxidizers (Fig. 1). The qPCR results of the *amoA* gene in the fractionated DNA showed that most comammox *Nitrospira* clade A members were labeled in freshwater microcosms but not in saline water microcosms (Fig. 3), whereas most AOA and AOB populations were labeled in both freshwater and saline water microcosms. In ^12^CO_2_ treatments, the relative abundances of AOA, AOB, and comammox *Nitrospira* peaked in fractions 12, 11, and 10, respectively, under both salinity conditions (Fig. 3). In the ^13^CO_2_-labeled freshwater microcosms, the peak of the comammox *Nitrospira* clade A communities shifted to heavy fractions, with buoyant densities of approximately 1.716 g/mL (fraction 9); however, this shift was not observed in the corresponding saline water microcosms. AOB populations in ^13^CO_2_-labeled microcosms shifted more under freshwater conditions (to fraction 9) than under saline water conditions (to fraction 10) (Fig. 3), revealing that AOB was more active in freshwater microcosms than in saline water microcosms. Regarding the AOA population, the peak shifted to a heavy fraction (fraction 10), with buoyant densities of 1.707–1.708 g/mL, both in the ^13^CO_2_-labeled freshwater and saline water microcosms.

### Phylogenetic analysis of ^13^C-labeled ammonia oxidizers

To determine the phylotypes of active ammonia oxidizers*,* the heavy fractions (fractions 8 and 9) of ^13^C-labeled microcosms (13 days) were amplified using specific primers for the comammox *Nitrospira amoA* gene and a universal primer set for the 16S rRNA gene.

**Fig 4:**
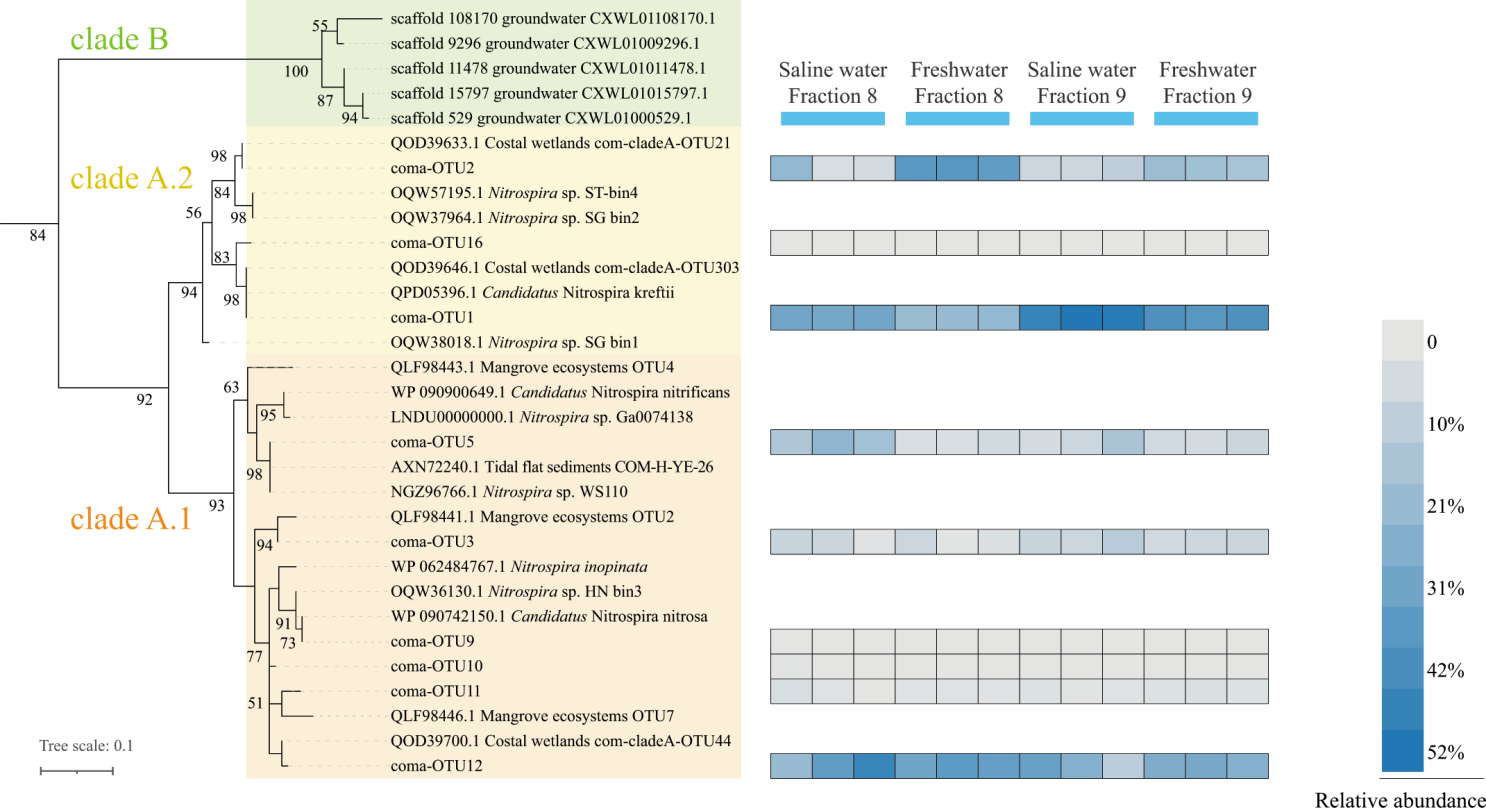
Phylogenetic analysis of the translated amino acid sequences of comammox *Nitrospira amoA* gene. Comammox *Nitrospira amoA* genes were amplified from the heavy fractions (fractions 8 and 9) of ^13^CO_2_-treated 13 days DNA-SIP microcosms incubated under freshwater and saline water conditions. The AmoA protein of *Nitrosomonas mobilis* (GenBank accession number AF287297.1) served as the outgroup of the tree. The green, yellow, and orange shading indicate comammox *Nitrospira* clades B, A.2, and A.1, respectively. Every treatment had three repeats. The color depth in each cell indicates the relative abundance of the corresponding OTU among all comammox *Nitrospira amoA* gene sequences in the indicated fraction. OTU, operational taxonomic unit.

Phylogenetic analysis (Fig. 4) revealed the diversity of comammox *Nitrospira* clade A in the heavy fractions, most of which were affiliated with clade A.2. The phylotypes of ^13^C-labeled OTUs were similar in the freshwater and saline water microcosms, but their relative abundances were different. Coma-OTU1 was the most dominant OTU in the heavy fractions and was close to the *amoA* gene of *Candidatus* Nitrospira kreftii in clade A.2. The relative abundance of coma-OTU1 in the heavy fractions of saline water microcosms (30±0.26% in fraction 8, 49 ± 2.6% in fraction 9) was higher than that in freshwater microcosms (21 ± 0.67% in fraction 8, 39 ± 1.4% in fraction 9). Coma-OTU2 in clade A.2 was two- and fourfold higher in fractions 8 and 9, respectively, of the freshwater microcosms than in those of saline water microcosms. Another main phylotype was coma-OTU12 in clade A.1, which was present in both the freshwater and saline water microcosms. These three OTUs also showed high identification with comammox *Nitrospira* OTUs detected in samples from coastal wetlands (13) (Fig. 4).

Active AOB and AOA were identified from DNA-SIP fractions 8 and 9 based on the analysis of 16S rRNA gene amplicon. The AOB phylogenetic tree revealed that the major ^13^C-labeled phylotype was AOB-OTU1, which is a sister clade of *Nitrosomonas aestuarii* (Fig. S4). The relative abundance of AOB-OTU1 was slightly higher in the heavy fractions of the saline water microcosms (91 ± 2.1%) than in those of the freshwater microcosms (73 ± 3.5%) (Fig. S4). Another AOB genus, *Nitrosospira*, had a relatively low abundance (≤ 0.01%) in heavy fractions. The phylogenetic tree of AOA showed that AOA-OTU3 was the most abundant phylotype and distantly affiliated with the 16S rRNA gene of the identified AOA (Fig. S5). AOA-OTU3 was more abundant in the freshwater microcosms (81 ± 1.2% in fraction 8, 84 ± 0.3% in fraction 9) than in the saline water microcosms (53 ± 18% in fraction 8, 63 ± 11% in fraction 9). Other major AOA OTUs clustered with the *Nitrosarchaeum*, *Nitrosopumilus*, or *Nitrosopelagicus* genera.

### Metagenome analysis of ^13^CO_2_-labeled ammonia oxidizers

A higher relative abundance of targeted microbes helps in binning them from the metagenome data. Hence, DNA-SIP freshwater microcosms were incubated for 18 days to increase the relative abundance of comammox *Nitrospira* in the heavy fractions (fractions 7–9).

Five comammox *Nitrospira* MAGs and eight NOB *Nitrospira* MAGs were assembled from the metagenome data (Table S2). High-quality MAGs (> 40% completeness and < 5% contamination) were used for the phylogenetic analysis of 71 housekeeping genes (Fig. S6). In the phylogenetic tree, *Nitrospira* bin98 and *Nitrospira* bin457 clustered with comammox *Ca*. *N. kreftii* and *Nitrospira* sp. ST-bin4, respectively. In *Nitrospira* bin98, an *amoCAB* gene cluster shared high sequence similarity with *Ca*. *N. kreftii* (100%, 99%, and 94% identity in terms of the protein-coding sequences of AmoA, AmoB, and AmoC, respectively), as shown in Table 1. The AmoA amino acid sequence of *Nitrospira* bin98 shared 100% identity with coma-OTU1. The AmoA protein of *Nitrospira* bin457 shared 100% identity with coma-OTU2, and the AmoA and AmoB proteins of *Nitrospira* bin457 shared 95.4% and 91.2% identity with that of *Nitrospira* sp. ST-bin4. Four bins of strict NOB *Nitrospira* were affiliated with sublineage II, and one bin was affiliated with sublineage IV (Fig. S6).

**TABLE 1:** Genome characteristics of comammox *Nitrospira*-like metagenome-assembled genomes (MAGs) closely related to coma-OTU1 and coma-OTU2, respectively

Characteristic	*Nitrospira* bin98	*Nitrospira* bin457
Completeness	93.12%	69.30%
Contamination	2.78%	0%
Clade	A	A
*amoA* gene	+^*a*^	+
*amoB* gene	+	+
*amoC* gene	+	−
*hao* gene	+	−

^*a*^ “+”: The indicated gene was recovered from the corresponding MAG; “−”: The indicated gene was not recovered from corresponding MAG.

**Fig 5:**
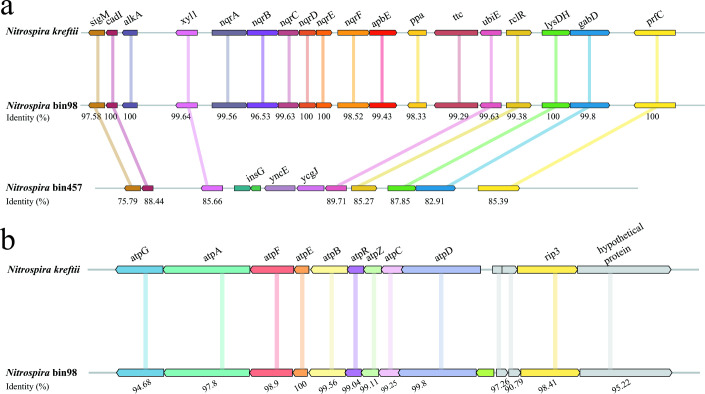
Na^+^-NQR (**a**) and N-ATP (**b**) loci in *Ca*. Nitrospira kreftii, *Nitrospira* bin98, and *Nitrospira* bin457. The amino acid identities (%) shared with *Ca*. Nitrospira kreftii are shown. The colored lines indicate the homologous genes in MAGs of comammox *Nitrospira*. The genes and noncoding regions are drawn to scale.

Gene clusters of N-type ATPase (N-*atp*) and sodium-pumping complex I (Na^+^-*nqr*) were identified in *Nitrospira* bin98, and these clusters matched those of *Ca*. *N. kreftii* (Fig. 5) and can be found in *Ca*. *Nitrospira alkalitolerans* (56). In *Nitrospira* bin457, although genes near the Na^+^-*nqr* gene cluster were identified, the Na^+^-*nqr* gene itself was not detected. Except for these two gene clusters, *Nitrospira* bin98 contains genes related to Na^+^:H^+^ antiporter, glycine betaine transporter, glutamate, and trehalose synthase (Data set 1), which were considered to be genes related to salt tolerance.

Based on our analysis of *amoA* gene reads retrieved from the freshwater metagenome data (Table S3), the three heavy fractions contained higher abundances of comammox *Nitrospira* than AOA and AOB. Clade A.2-related *amoA* gene reads (with >90% identity) were most abundant in fractions 7–9. The *amoA* gene reads of AOB mainly belonged to *Nitrosomonas* cluster 6b and were present at a much lower abundance than that found with comammox *Nitrospira*. Surprisingly, the *amoA* gene reads of AOA were not found in that metagenome data, even with a reduced identity threshold of 41%. Additionally, no AOA MAG was assembled from our metagenome data.

### PAR determinations

**Fig 6:**
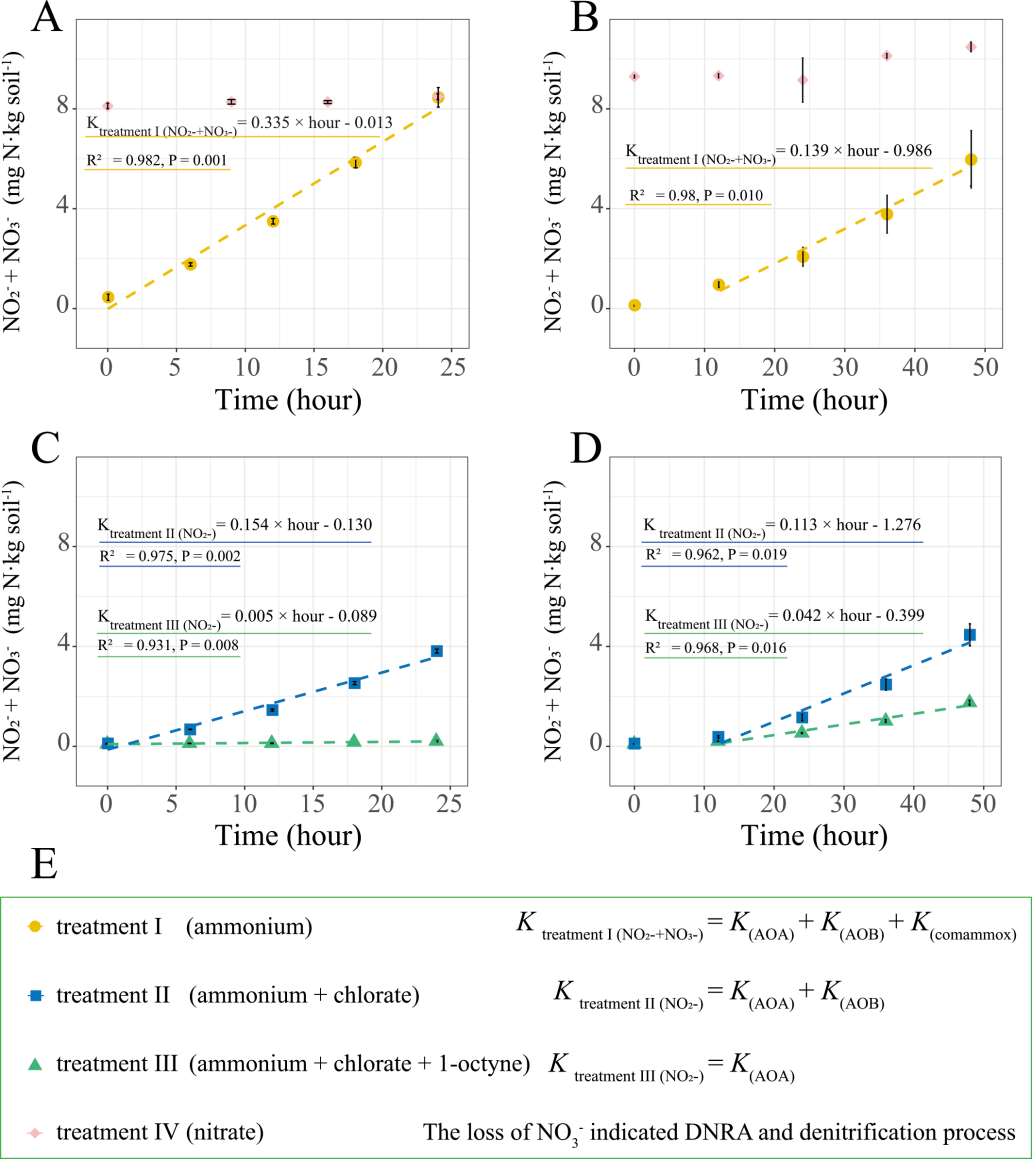
NO_2_^−^ and NO_3_^−^ concentrations in samples following treatments I–IV. The NO_2_^−^ and NO_3_^−^ concentrations were determined to assess the potential ammonium-oxidation rates (PARs) of AOA, AOB, and comammox *Nitrospira*. Ammonium-oxidation activity of treatments I and IV in freshwater condition (**A**) and saline water condition (**B**). Ammonium-oxidation activity of treatments II and III in freshwater water (**C**) and saline water (**D**) conditions. The formula calculated activity of AOA, AOB, and comammox *Nitrospira* are also listed (**E**). The dotted lines are linear-fitting curves. For linear-fitting curves in (**B**) and (**D**), activities with saline water conditions at 0–12 h were not included in the calculation due to the low activity for the adaptation to high salinity in the early stage. The error bars represent standard deviations. Abbreviations: AOA, ammonia-oxidizing archaea; AOB, ammonia-oxidizing bacteria; DNRA, dissimilatory nitrate reduction to ammonium.

Chlorate and 1-octyne were used to determine the PARs of different ammonia oxidizers under four different treatments (Fig. 1 and 6).

The PAR of comammox *Nitrospira* was much higher under freshwater conditions (4.37 ± 0.53 mg N·day^–1^·kg soil^–1^) than that under saline water (0.60 ± 0.94 mg N·day^–1^·kg soil^–1^) (Fig. 6). The PAR of AOB under saline water conditions (1.74 ± 0.31 mg N·day^–1^·kg soil^–1^) was approximately half that under freshwater conditions (3.58 ± 0.13 mg N·day^–1^·kg soil^–1^). The PAR of AOA under saline water conditions was 1.02 ± 0.06 mg N·day^–1^·kg soil^–1^, whereas under freshwater conditions, PAR was negligible. The effects of dissimilatory nitrate reduction to ammonium (DNRA) and denitrification processes were ignored in this study because the nitrate concentration did not decrease during treatment IV.

In the freshwater microcosms, comammox *Nitrospira,* AOB, and AOA contributed 54%, 44%, and 2% to the total ammonia oxidation, respectively(Table 2). The contribution of comammox *Nitrospira* decreased to 18% under saline water conditions and was much lower than that of AOB (52%) and AOA (30%). These results indicate that salinity greatly affected the activities of ammonia oxidizers, especially for comammox *Nitrospira* and AOA.

**TABLE 2:** Potential ammonium-oxidation rates (PARs) of AOA, AOB, and comammox *Nitrospira* and their contributions to ammonia oxidation under freshwater and saline water conditions

		Freshwater condition	Saline water condition
PARs under different treatments (mg N·day^–1^·kg soil^–1^)	Treatment I	8.06 ± 0.44	3.36 ± 0.69
	Treatment II	3.70 ± 0.10	2.76 ± 0.25
	Treatment III	0.12 ± 0.04	1.02 ± 0.06
The estimated contribution of different nitrifiers	AOA	0.12 ± 0.04 (2%)	1.02 ± 0.06 (30%)
	AOB	3.58 ± 0.13 (44%)	1.74 ± 0.31 (52%)
	Comammox *Nitrospira*	4.37 ± 0.53 (54%)	0.60 ± 0.94 (18%)

## DISCUSSION

The broad distribution of comammox *Nitrospira* has been detected in coastal ecosystems, yet their relationship with salinity remains controversial (8, 13, 57). Here, we demonstrated that increased salinity decreased the contribution of total comammox *Nitrospira* to nitrification in intertidal sediment microcosms while part of comammox *Nitrospira* still survived in saline water conditions.

Previous studies on comammox *Nitrospira* have focused on their relative or absolute abundances in different ecosystems based on metagenome or qPCR methods (7, 10, 13). However, activity-based analyses of the contribution of comammox *Nitrospira* to nitrification have been reported in only a few studies (16, 17). In this study, we conducted microcosm experiments, coupled with DNA-SIP analysis and activity tests, to better define the activity of comammox *Nitrospira* in intertidal sediments.

Salinity is generally assumed to be important for the community structures, abundances, and activities of canonical ammonia oxidizers in coastal ecosystems (58, 59). Although numerous findings have revealed that the distributions of AOA and AOB (60) or comammox *Nitrospira* (12, 30) correlated with salinity in different coastal environments, the results are inconsistent. Such discrepancies may be attributed to other environmental factors obscuring the effects of salinity (61). In our microcosm experiments, salinity was the only variable, and the abundance of different ammonia oxidizers was of the same order of magnitude in the original sediment; hence, the impact of salinity on ammonia oxidizers could be clearly assessed. Under saline water conditions, which have the same salinity as seawater, the abundance of comammox *Nitrospira* was significantly lower than under freshwater conditions (Fig. 2). The low abundance of comammox *Nitrospira* at high salinities is consistent with affiliation with *Nitrospira* sublineage II (3, 4), which is frequently observed in low salinity environments such as freshwater (62) and terrestrial habitats (63). Unlike *Nitrospira* sublineage IV (56), which is mainly distributed in marine and estuarine environments (64, 65), most strains in sublineage II lack a complete set of genes for cation homeostasis and osmotic stress-defense mechanisms (66).

The DNA-SIP results supported the higher activity of comammox *Nitrospira* under freshwater conditions than under saline water conditions (Fig. 3) and revealed that different comammox *Nitrospira* phylotypes were differently affected by salinity (Fig. 4). Coma-OTU2, the primary phylotype of comammox *Nitrospira* clade A.2 detected in heavy fractions, was dominant under freshwater conditions (approximately 38% and 18% in fractions 8 and 9, respectively). Unlike coma-OTU2, which was only prevalent in freshwater conditions, a high proportion of coma-OTU1 was found under both freshwater and saline water conditions. We assembled that the MAGs matched to coma-OTU1 and coma-OTU2 were *Nitrospira* bin98 and *Nitrospira* bin457, respectively (Fig. S6; Table 1). *Nitrospira* bin98 possesses several genes to adapt to saline environments (Data set 1), unlike *Nitrospira* bin457. N-ATPase (Na^+^-translocating N-ATPase) and sodium-pumping complex I (Na^+^-translocating NADH-quinone reductase, Na^+^-NQR) (Fig. 5) were identified in *Nitrospira* bin98 and haloalkaline *Ca*. *N. alkalitolerans* (56) in *Nitrospira* sublineage IV, indicating their role in mitigating the toxicity associated with excessive Na^+^. They are thought to participate in Na^+^ export when synthesizing ATP (67) and ubiquinol (68). NhaA, nhaB, nhaR, and nhaP, retrieved from *Nitrospira* bin98, are members of Na^+^:H^+^ antiporter group (69), which is widely distributed in all living microorganisms (70), involved in restoring the growth of microorganisms (71) and maintaining the Na^+^ homeostasis (72) when the external salt concentration is high. Generally, K^+^, rather than Na^+^, is accumulated intracellularly to balance the high external Na^+^ concentration in halophilic bacteria (73) because of the toxicity of high Na^+^ to cells (74). Although the genes encoding Trk protein, considered the key enzyme for K^+^ uptake (75), cannot be found in *Nitrospira* bin98, the NaCl-mediated K^+^-transporting gene, kdpFABC (76), was retrieved from the bin. Additionally, *Nitrospira* bin98 possesses genes encoding proteins for compatible solute synthesis and import, which is another important mechanism microorganisms use to regulate intracellular osmotic pressure (77). Glycine betaine is a compatible solute that improves the salt tolerance of bacteria and archaea (78). *Nitrospira* bin98 contains protein-coding *opu* genes for glycine betaine import (79), which are also assembled in marine nitrifiers *Nitrosococcus halophilus* (80) and *Nitrospina gracilis* (81). In addition, *Nitrospira* bin98 has two copies of the trehalose synthase gene found in other nitrifiers like *Nitrosococcus wardiae* (82) and *Nitrosomonas halophila* (UniProt: A0A1H3EVT1), enabling synthesis of compatible solute trehalose from maltose (83, 84). However, these compatible solute synthesis/import and various Na^+^ exporting genes cannot be found in *Nitrospira* bin457 (Data set 1), which may help explain the survival of coma-OTU1 (*Nitrospira* bin98) rather than coma-OTU2 (*Nitrospira* bin457) under saline water conditions. Notably, the heterogeneity in comammox *Nitrospira* members, such as that in *Nitrospira* bin98 and bin457, has been previously reported (8, 12). The correlation between OTUs and salinity differed, even within the same clade A.1 (or A.2). We speculate that the previously reported correlation between comammox *Nitrospira* and salinity might have been influenced by the presence or absence of genes that facilitate adaptation to saline environments in dominant OTUs. In addition, coma-OTU2 was identical to previously reported comammox *Nitrospira* OTU21 (QOD39633.1) and AmoA of MAG *Nitrospira* YR-XLD (MBH0181332.1), which were a representative OTU in Yangtze River estuarine tidal flat wetlands (13) and predominant comammox *Nitrospira* in Yangtze River water (85), respectively. This implies that coma-OTU2 may be a common type in aquatic systems. Unlike coma-OTU2, OTUs or AmoA proteins, similar to coma-OTU1, were retrieved from different environments, such as flat tidal sediments (AXN72286.1 and UPN65918.1), wastewater treatment plant (QPD05396.1 and VWF18671.1), peritidal stromatolite (NJN71137.1), and reservoir area stream (USE40431.1). This difference indicates that coma-OTU1 may have a broader distribution and enhanced adaptation than coma-OTU2. Furthermore, considering that we detected coma-OTU1 in the heavy fractions of saline water samples in this study and previous observations of diverse comammox *Nitrospira amoA* genes in mangrove ecosystems with high salinity (30), some types of comammox *Nitrospira* may be salt-tolerant and survive under certain saline water ecosystems. More environmental samples with different salinities should be studied in the future to gain a more comprehensive understanding of the relationships between salinity and different types of comammox *Nitrospira*.

Under freshwater conditions, comammox *Nitrospira* contributed to over half of the total ammonia oxidation activity, whereas comammox *Nitrospira* contributed to only 18% under saline water conditions (Fig. 6 and Table 2). These findings are consistent with our results of microcosm DNA-SIP-based qPCR analysis (Fig. 3) that only part of comammox *Nitrospira* was active in saline water conditions. The mechanisms by which salinity inhibits the activity of ammonia oxidizers, except for the direct impact on increased osmotic pressure discussed earlier, include the decrease of ammonium (86) and dissolved oxygen (87) in coastal ecosystems, which are side effects of increased salinity and are important for the activity of AOA and AOB (88, 89). Nonetheless, we did not regard the decrease of ammonium or dissolved oxygen as the reason for the decreased activity of comammox *Nitrospira*. Comammox *Nitrospira* has a high affinity for ammonia, which allows them to grow in environments with low ammonia concentrations (90), and contains genes that might allow efficient growth at low oxygen concentrations (91). Additionally, considering the high activity of comammox *Nitrospira* in freshwater conditions, comammox *Nitrospira* may be the main contributor to nitrification in intertidal zones due to the low salinity of intertidal sediments in summer with high river-runoff levels (34). Comammox *Nitrospira* also have been reported to contribute highly to the nitrification process in plateau wetlands (92); therefore, the contributions of comammox *Nitrospira* in different terrestrial ecosystems should be considered.

The PAR experiments also revealed that AOB was the dominant contributor to nitrification (Table 2) under saline water conditions. Phylogenetic analysis of the 16S rRNA gene sequences from the heavy fraction of DNA-SIP microcosms showed that the active AOB were grouped in *Nitrosomonas* cluster 6b (Fig. S4), which includes *Nitrosomonas marina* (93) and *Nitrosomonas aestuarii* (94). This group is common in both coastal and marine environments (95, 96). The activity of AOA was much lower than those of comammox *Nitrospira* and AOB under freshwater conditions (Table 2). The observation that the *amoA* gene of AOA was not detected in the metagenome data of heavy fractions of freshwater DNA-SIP samples (Table S3) also suggests that AOA may contribute relatively little to ammonia oxidation under freshwater conditions. During microcosm incubation, nitrite transiently accumulates in high salinity conditions, and this phenomenon was also observed in other coastal ecosystems (97, 98). AOB can cause nitrite accumulation by decoupling ammonia oxidation from nitrite oxidation due to their fast enzyme (99) and growth (100) kinetics. In addition, the relative abundance of AOB increases with higher salinity in microcosms experiments (Fig. S2), indicating its important role under high salinity conditions.

As an inhibitor of NOB (101, 102), chlorate is normally used for potential nitrification rate analysis of AOA and AOB (19, 103). However, ammonia oxidation by comammox *Nitrospira* is also inhibited by chlorate (22) because of the toxicity of chlorite, produced from the reduction of chlorate by nitrite oxidoreductase, which drives nitrite oxidation during complete ammonia oxidation (21). Therefore, in this study, chlorate was used as a selective inhibitor of comammox *Nitrospira* to distinguish it from canonical ammonia oxidizers (i.e., AOA and AOB) as demonstrated previously (21) (i.e., treatment II), and a combination of chlorate and 1-octyne was used to inhibit comammox *Nitrospira* and AOB (treatment III) (Fig. 6). Wang *et al*. (57) reported a method to differentiate the potential comammox activity of comammox *Nitrospira* from the PARs of AOA and AOB using chlorate and 1-octyne as inhibitors, wherein the potential comammox activity was calculated by subtracting the nitrate-production rate by nitrite oxidation from the total nitrate-production rate (57). However, nitrite leakage by *Nitrospira inopinata* (4) and efficient nitrite oxidation by *Ca*. *N. kreftii* (104) indicated that comammox *Nitrospira* releases a portion of the nitrite produced via ammonia oxidation from their cells and oxidizes extracellular nitrite to nitrate. This finding indicates that when only considering the complete ammonia oxidation process as per the method of Wang *et al*. (57), the ammonia oxidation activity of comammox *Nitrospira* may be underestimated if nitrite is released by the organism and overestimated if external nitrite is oxidized by comammox *Nitrospira*.

Here, we investigated the effect of salinity on the nitrification activity of comammox *Nitrospira*, AOB, and AOA in intertidal sediments. Our results suggested that salinity is an important factor that affects the abundance, community structure, and activity of comammox *Nitrospira* from Yangtze River intertidal flats. Meanwhile, since we selected a sample containing similar amounts of different types of ammonia oxidizers, further investigations from different coastal environments, especially with frequent fluctuations in salinity levels, remain warranted to generalize and consolidate the conception that salinity exerts an important control on comammox *Nitrospira* and that salinity tolerance differs between different comammox *Nitrospira* genotypes. Since the discovery of comammox *Nitrospira* in coastal ecosystems, several studies have reported contradicting correlations between comammox *Nitrospira* and salinity (8, 12, 13). Our findings revealed that various *Nitrospira* OTUs have different tolerances to salinity, which could explain why the two specific OTUs have contrasting correlations with salinity. Furthermore, comammox *Nitrospira* may contribute to the reduction of nitrite accumulation caused by AOB nitrification decoupling in coastal environments.

## Data Availability

The data sets supporting the conclusions of this article are available in National Omics Data Encyclopedia (https://www.biosino.org) repository, OEP002773 (https://www.biosino.org/node/project/detail/OEP002773). It contains 16S rRNA gene amplicons of total bacteria, *amoA* gene amplicons of comammox *Nitrospira*, and raw metagenome sequences. Analytical scripts can be found at https://github.com/traminer23333.

## SUPPLEMENTAL MATERIAL

Fig. S1 msystems.01026-22-s0001.tif Changes in the nitrate (a) and nitrite (b) concentrations during microcosm incubations with different salinities. The error bars indicate the standard errors of triplicate samples.

Fig. S2 msystems.01026-22-s0002.tif 16S rRNA gene abundances. Changes in the relative abundances of the 16S rRNA gene of AOA (*Nitrosopumilus* and *Nitrosoarchaeum*), AOB (*Nitrosomonas* and *Nitrosospira*), and *Nitrospira* in microcosms incubated for 18 days under different salinity conditions.

Fig. S3 msystems.01026-22-s0003.tif Relative abundances of strict NOB and comammox bacteria. Abundances were determined by studying 16S rRNA gene amplicons in microcosms incubated for 18 days under different salinity conditions.

Fig. S4 msystems.01026-22-s0004.tif Phylogenetic analysis of AOB 16S rRNA gene amplicons. The AOB 16S rRNA gene amplicons were retrieved from the heavy fractions (fractions 8 and 9) of 13-day DNA-SIP microcosms incubated with ^13^CO_2_ under freshwater and saline water conditions. The 16S rRNA gene of *Nitrosospira multiformis* (GenBank accession number AY123807.1) served as the outgroup for the phylogenetic tree. Every treatment had three repeats. The color depth indicates the relative abundance of the indicated OTU among all *Nitrosomonas* 16S rRNA gene sequences in the indicated fraction.

Fig. S5 msystems.01026-22-s0005.tif Phylogenetic analysis of AOA 16S rRNA gene amplicons. The AOA 16S rRNA gene amplicons were retrieved from the heavy fractions (fractions 8 and 9) of 13-day DNA-SIP microcosms incubated with ^13^CO_2_ under freshwater and saline water conditions. The 16S rRNA gene of *Nitrososphaera viennensis* (GenBank accession number NR_134097.1) served as the outgroup for the phylogenetic tree. Each treatment had three repeats. The color depth indicates the relative abundance of the indicated OTU among all AOA 16S rRNA gene sequences in the indicated fraction.

Fig. S6 msystems.01026-22-s0006.tif Phylogenomic mapping of *Nitrospira* MAGs. The *Nitrospira* MAGs were retrieved from the heavy fractions (fractions 7-9) of 18-day freshwater DNA-SIP microcosms, and *Nitrospira* genomes and MAGs deposited in NCBI, based on concatenation of the amino acid sequences of 71 housekeeping genes. The orange and green shading indicates comammox *Nitrospira* clade A and clade B clusters, respectively. Regarding MAGs assembled in this research, the purple and red text indicate cases where the corresponding bins were NOB *Nitrospira* and comammox *Nitrospira* clade A, respectively. Since portions of the published assemblies were not named, we refer to them with the designation “*Nitrospira* sp. (Whole-Genome Shotgun project number).” Abbreviation: MAGs, metagenome-assembled genomes.

Table S1 msystems.01026-22-s0007.tif Primers used in this study.

Table S2 msystems.01026-22-s0008.tif Genome characteristics of the retrieved *Nitrospira* MAGs.

Table S3 msystems.01026-22-s0009.tif Abundances of different *amoA* genes in the heavy-fraction metagenome data for DNA-SIP microcosm under freshwater conditions.

Data Set S1 msystems.01026-22-s0010.xlsx Comparison of proteins with predicted functions in adaptation to high salinity in *Nitrospira* genus.

ASM does not own the copyrights to Supplemental Material that may be linked to, or accessed through, an article. The authors have granted ASM a non-exclusive, world-wide license to publish the Supplemental Material files. Please contact the corresponding author directly for reuse.
