# Supplementary figures and images for: Salinity changes the nitrification activity and community composition of comammox Nitrospira in intertidal sediments of Yangtze River estuary
Source: mSystems. 2023 Jun 12;8(3):e01026-22. doi: 10.1128/msystems.01026-22 (PMC10308925; doi:10.1128/msystems.01026-22)

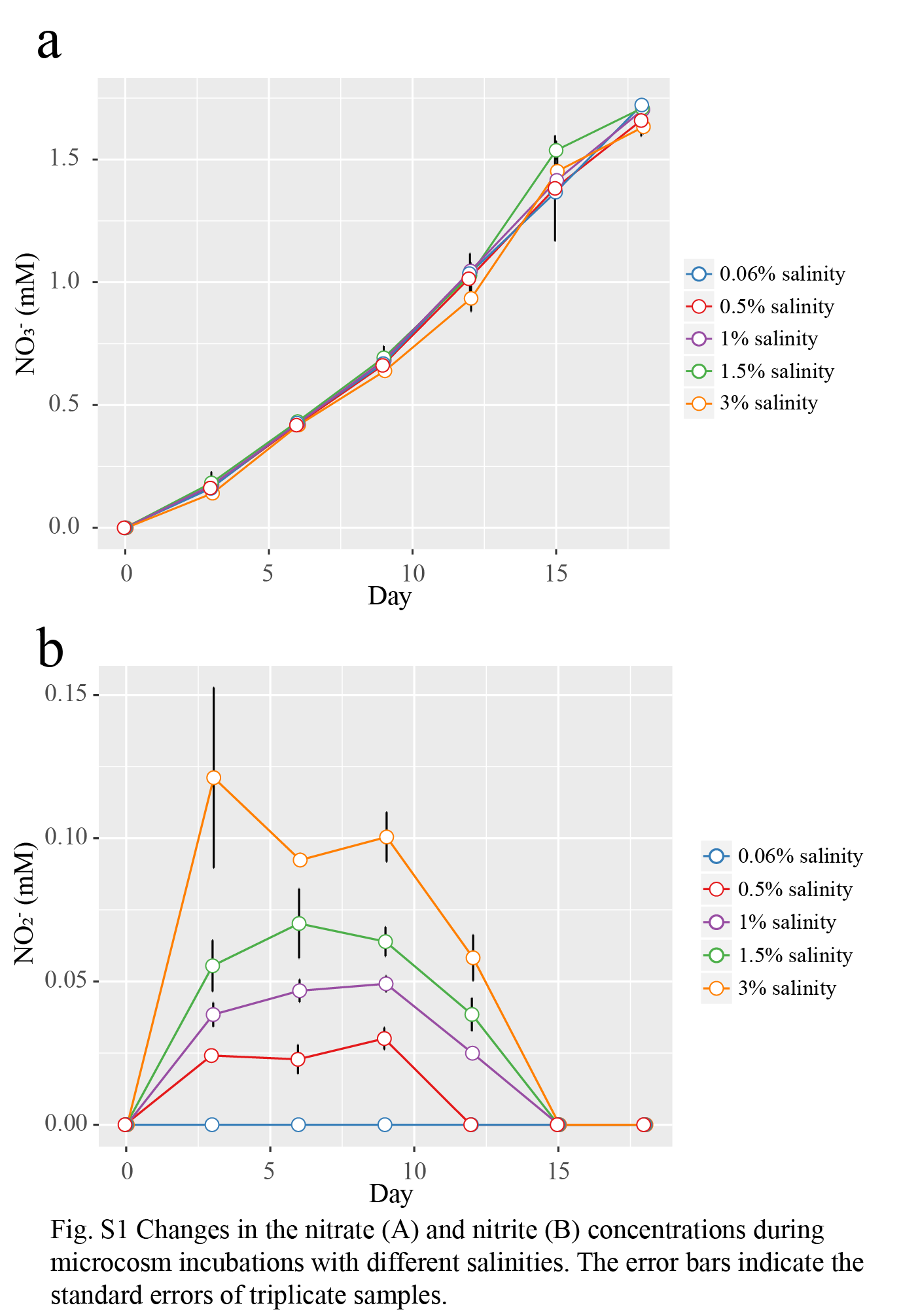

Supplement: Fig. S1 — Changes in the nitrate (a) and nitrite (b) concentrations during microcosm incubations with different salinities. The error bars indicate the standard errors of triplicate samples. [file msystems.01026-22-s0001.tif]

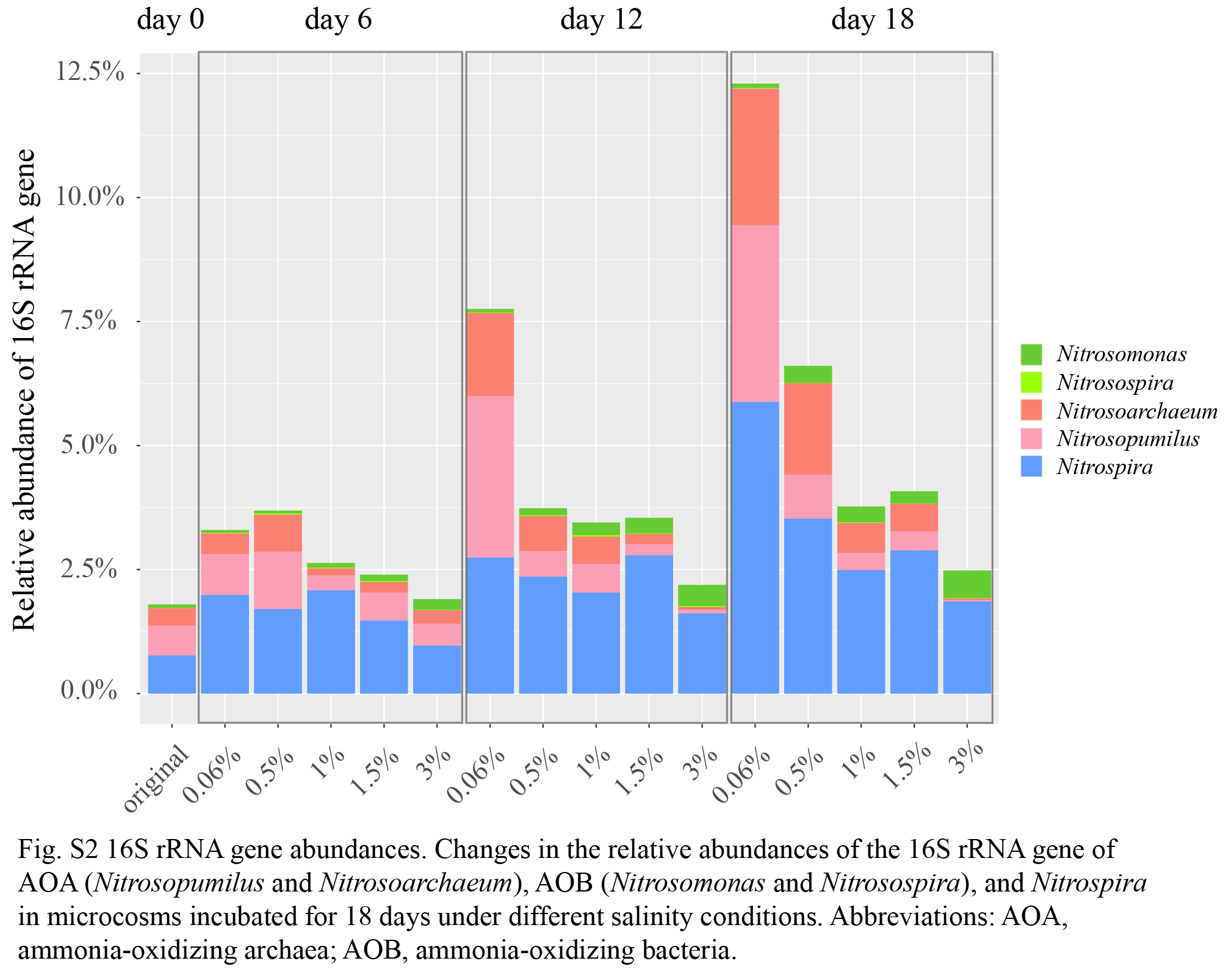

Supplement: Fig. S2 — 16S rRNA gene abundances. Changes in the relative abundances of the 16S rRNA gene of AOA (Nitrosopumilus and Nitrosoarchaeum), AOB (Nitrosomonas and Nitrosospira), and Nitrospira in microcosms incubated for 18 days under different salinity conditions. [file msystems.01026-22-s0002.tif]

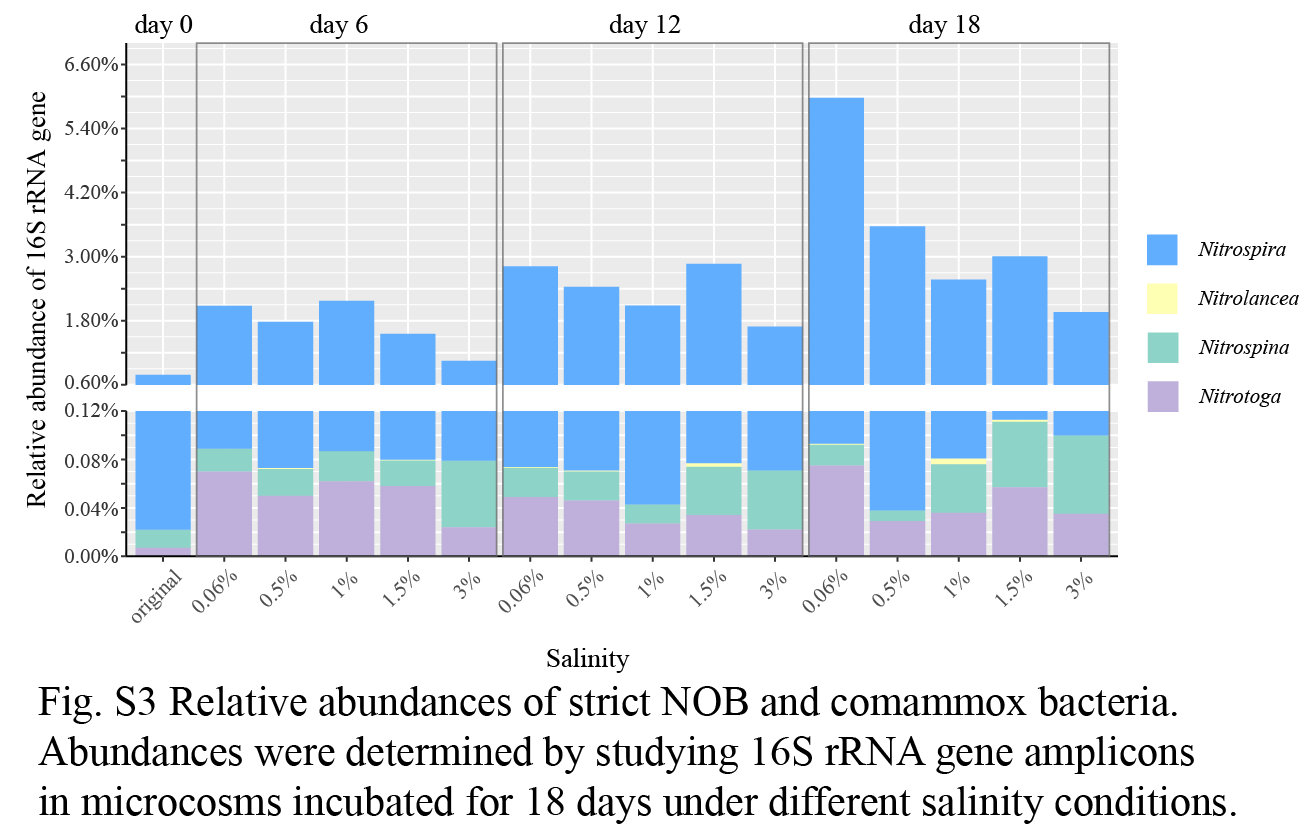

Supplement: Fig. S3 — Relative abundances of strict NOB and comammox bacteria. Abundances were determined by studying 16S rRNA gene amplicons in microcosms incubated for 18 days under different salinity conditions. [file msystems.01026-22-s0003.tif]

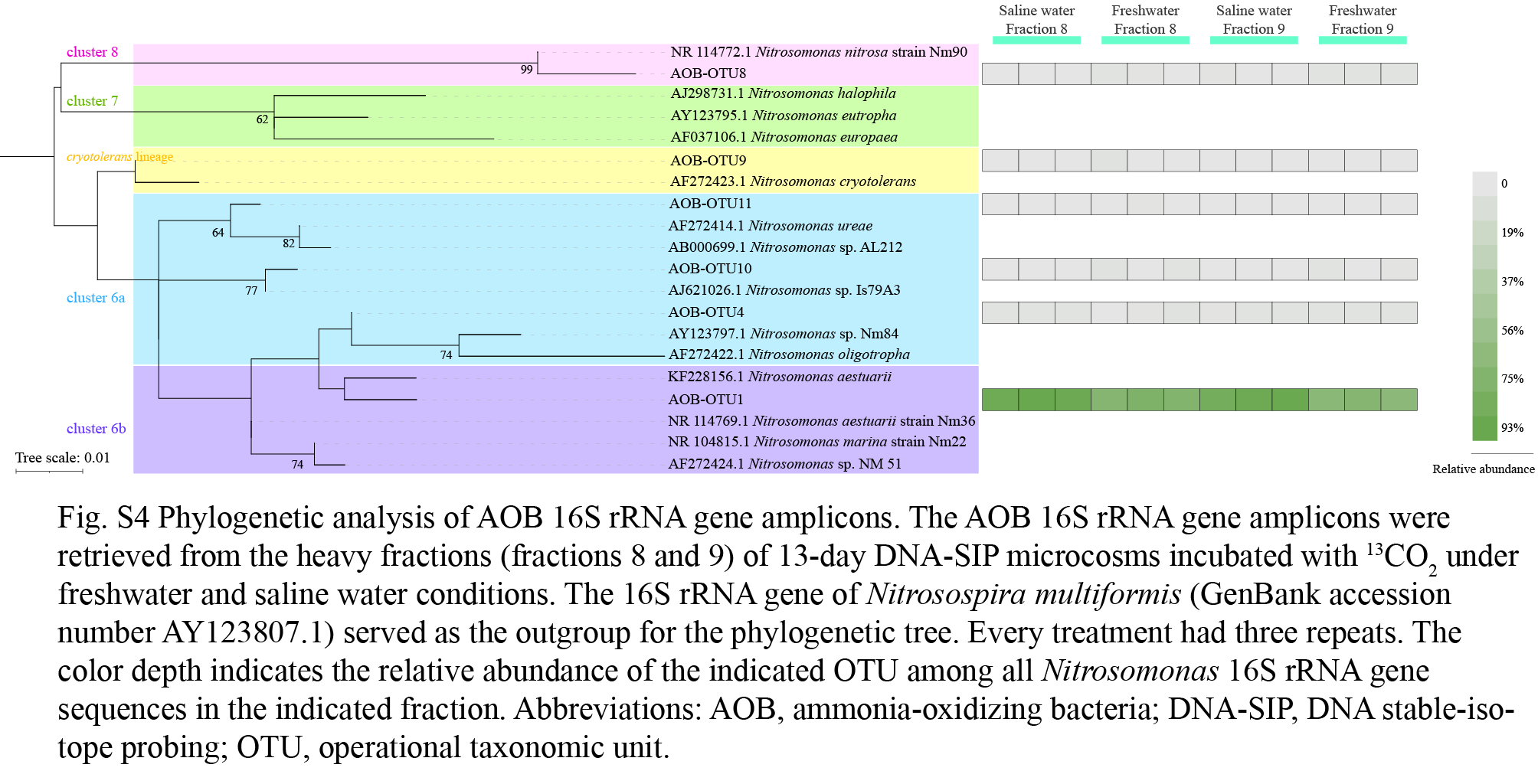

Supplement: Fig. S4 — Phylogenetic analysis of AOB 16S rRNA gene amplicons. The AOB 16S rRNA gene amplicons were retrieved from the heavy fractions (fractions 8 and 9) of 13-day DNA-SIP microcosms incubated with 13CO2 under freshwater and saline water conditions. The 16S rRNA gene of Nitrosospira multiformis (GenBank accession number AY123807.1) served as the outgroup for the phylogenetic tree. Every treatment had three repeats. The color depth indicates the relative abundance of the indicated OTU among all Nitrosomonas 16S rRNA gene sequences in the indicated fraction. [file msystems.01026-22-s0004.tif]

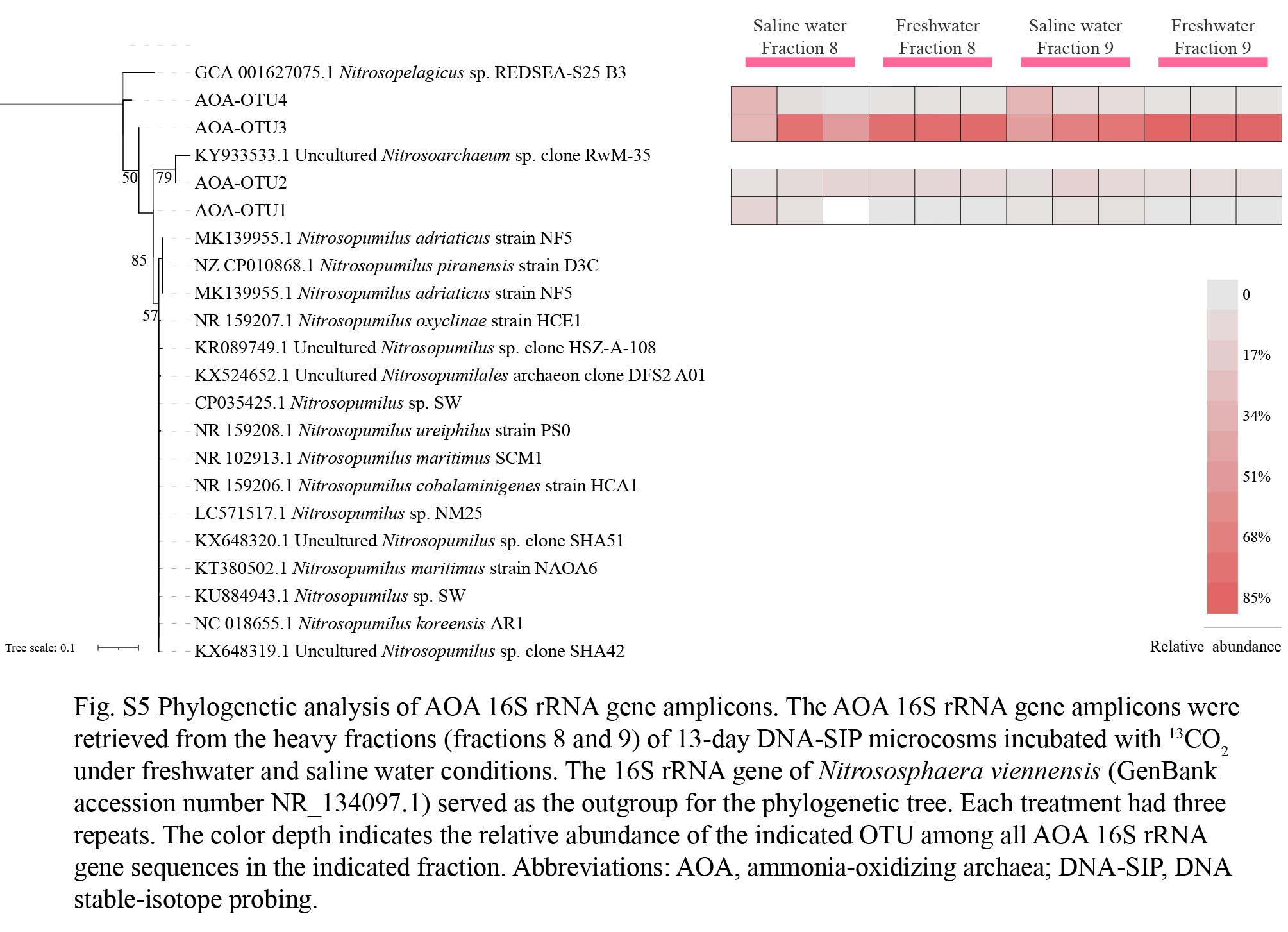

Supplement: Fig. S5 — Phylogenetic analysis of AOA 16S rRNA gene amplicons. The AOA 16S rRNA gene amplicons were retrieved from the heavy fractions (fractions 8 and 9) of 13-day DNA-SIP microcosms incubated with 13CO2 under freshwater and saline water conditions. The 16S rRNA gene of Nitrososphaera viennensis (GenBank accession number NR_134097.1) served as the outgroup for the phylogenetic tree. Each treatment had three repeats. The color depth indicates the relative abundance of the indicated OTU among all AOA 16S rRNA gene sequences in the indicated fraction. [file msystems.01026-22-s0005.tif]

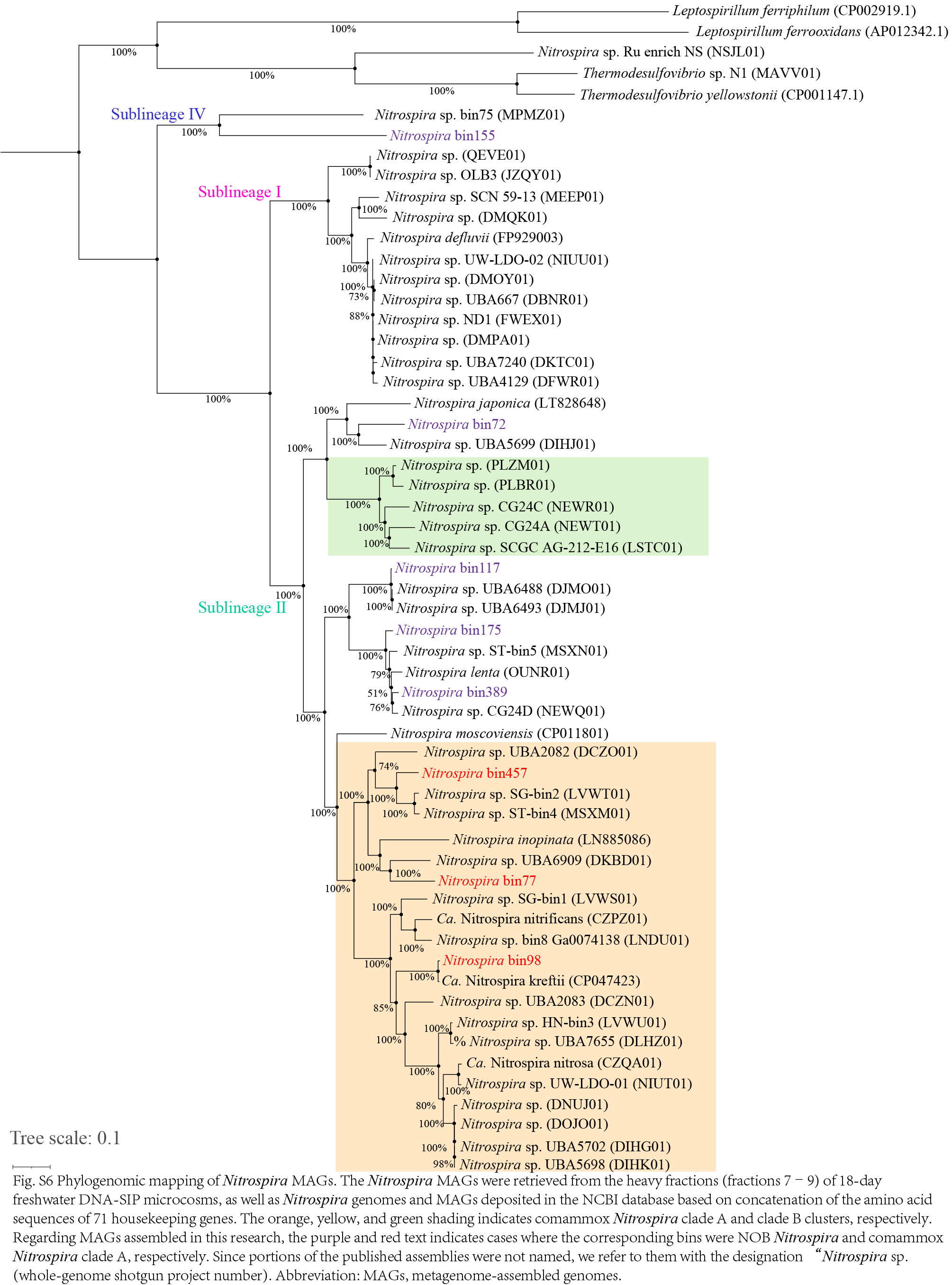

Supplement: Fig. S6 — Phylogenomic mapping of Nitrospira MAGs. The Nitrospira MAGs were retrieved from the heavy fractions (fractions 7-9) of 18-day freshwater DNA-SIP microcosms, and Nitrospira genomes and MAGs deposited in NCBI, based on concatenation of the amino acid sequences of 71 housekeeping genes. The orange and green shading indicates comammox Nitrospira clade A and clade B clusters, respectively. Regarding MAGs assembled in this research, the purple and red text indicate cases where the corresponding bins were NOB Nitrospira and comammox Nitrospira clade A, respectively. Since portions of the published assemblies were not named, we refer to them with the designation “Nitrospira sp. (Whole-Genome Shotgun project number).” Abbreviation: MAGs, metagenome-assembled genomes. [file msystems.01026-22-s0006.tif]

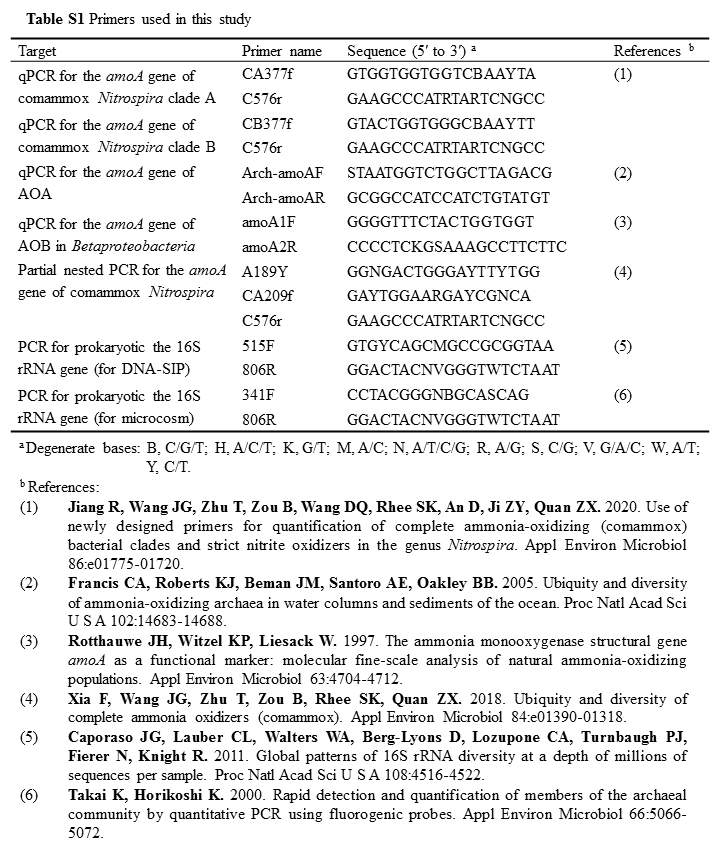

Supplement: Table S1 — Primers used in this study. [file msystems.01026-22-s0007.tif]

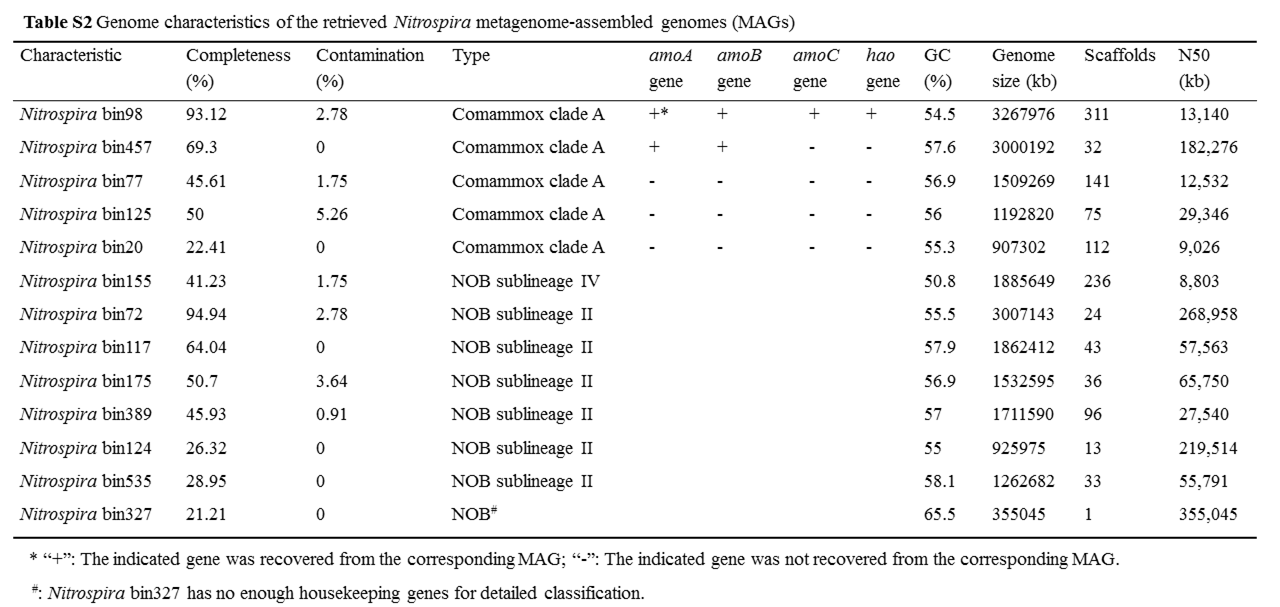

Supplement: Table S2 — Genome characteristics of the retrieved Nitrospira MAGs. [file msystems.01026-22-s0008.tif]

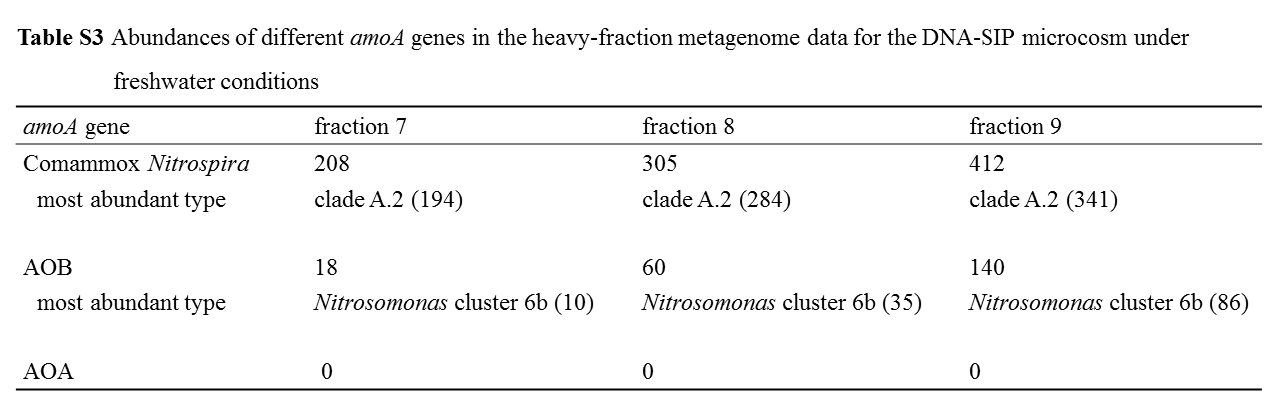

Supplement: Table S3 — Abundances of different amoA genes in the heavy-fraction metagenome data for DNA-SIP microcosm under freshwater conditions. [file msystems.01026-22-s0009.tif]
